# COVID‐19 is an emergent disease of aging

**DOI:** 10.1111/acel.13230

**Published:** 2020-10-01

**Authors:** Didac Santesmasses, José Pedro Castro, Aleksandr A. Zenin, Anastasia V. Shindyapina, Maxim V. Gerashchenko, Bohan Zhang, Csaba Kerepesi, Sun Hee Yim, Peter O. Fedichev, Vadim N. Gladyshev

**Affiliations:** ^1^ Division of Genetics, Department of Medicine Brigham and Women’s Hospital and Harvard Medical School Boston Massachusetts USA; ^2^ GERO PTE. LTD Singapore City Singapore; ^3^ Faculty of Bioengineering and Bioinformatics Lomonosov Moscow State University Moscow Russia; ^4^ Research Center for Molecular Mechanisms of Aging and Age Related Diseases Moscow Institute of Physics and Technology (National Research University) Dolgoprudny Russia

**Keywords:** age‐related diseases, COVID‐19, gene expression, lifespan, pneumonia, viral infection

## Abstract

COVID‐19 is an ongoing pandemic caused by the SARS‐CoV‐2 coronavirus that poses one of the greatest challenges to public health in recent years. SARS‐CoV‐2 is known to preferentially target older subjects and those with pre‐existing conditions, but the reason for this age dependence is unclear. Here, we found that the case fatality rate for COVID‐19 grows exponentially with age in all countries tested, with the doubling time approaching that of all‐cause human mortality. In addition, men and those with multiple age‐related diseases are characterized by increased mortality. Moreover, similar mortality patterns were found for all‐cause pneumonia. We further report that the gene expression of ACE2, the SARS‐CoV‐2 receptor, grows in the lung with age, except for subjects on a ventilator. Together, our findings establish COVID‐19 as an emergent disease of aging, and age and age‐related diseases as its major risk factors. In turn, this suggests that COVID‐19, and deadly respiratory diseases in general, may be targeted, in addition to antiviral approaches, by approaches that target the aging process.

## Graphical abstract

This study establishes COVID‐19 as an emergent disease of aging, based on (a) an exponential growth of the COVID‐19 mortality rate with age, (b) the COVID‐19 mortality rate doubling time approaching that of all‐cause human mortality, (c) higher mortality in men than in women, (d) strong association with pre‐existing age‐related diseases, and (e) COVID‐19 being a subset of all‐cause pneumonia, which we find itself is a disease of aging.

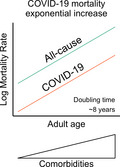

## 1 INTRODUCTION

Our society faces unprecedented challenges as the global pandemic of the coronavirus disease 2019 (COVID‐19) spreads around the world, with more than 12.5 million cases and 550,000 deaths reported (“WHO”, 2020). The disease is caused by an enveloped single‐stranded positive RNA virus named severe acute respiratory syndrome coronavirus 2 or SARS‐CoV‐2 (Wu, Zhao, et al., 2020). In contrast to other coronaviruses, SARS‐CoV‐2 has the ability to infiltrate the lower respiratory tract resulting in severe lung damage and a high rate of deaths from pneumonia (Zhu et al., 2020).

Older subjects, men, and those with pre‐existing conditions such as hypertension, diabetes, cancer, heart failure, and chronic obstructive pulmonary disease are more prevalent among hospitalized COVID‐19 patients (Richardson et al., 2020; Wang et al., 2020). Clinical risk factors for COVID‐19‐related deaths have been identified using a very large cohort (Williamson et al., 2020). The most common comorbidities have age as a risk factor and have been described in recent years as age‐related diseases. The COVID‐19 case fatality rate (CFR), that is, the quotient of deaths to confirmed infections, was shown to be lower in patients below 60 years old (1.4% [0.4–3.5]) compared to those who were 60 years or older (4.5% [1.8–11.1]) (Verity et al., 2020). However, the reason why older patients and those with pre‐existing conditions display a higher risk for COVID‐19 is currently unknown.

SARS‐CoV‐2 is a respiratory virus mainly transmitted through air droplets that initiates infection in the lung. The severity of the respiratory illness might be related to age‐associated changes in the physical properties of the lung and the decline of the immune function, known as immunosenescence. The lung employs mechanical defenses such as cough, the barrier function of the mucus and epithelium, and mucociliary clearance, which, syncronized with the innate immune system, help to clear aspirated or inhaled substances, including infectious agents (Meyer, 2001). However, these and other concerted actions are known to decrease with aging.

In general, the idea that older people are more susceptible to infections is not new. In fact, it has been reported that up to one third of deaths in the elderly is a result of infectious diseases (Kline & Bowdish, 2016). Persistent viral infections may also trigger monoclonal expansion of T cells, which over the lifetime results in poor variability of memory T cells. In turn, this eventually drives immune exhaustion due to the decline in T‐cell diversity (Brunner, Herndler‐Brandstetter, Weinberger, & Grubeck‐Loebenstein, 2011), a critical problem when facing novel threats such as SARS‐CoV‐2.

An additional feature that characterizes the severe cases of COVID‐19 is the elevated levels of inflammation that can compromise lung tissue integrity and function, leading to pneumonia. Remarkably, accumulated and exhausted T cells secrete preferentially pro‐inflammatory cytokines such as IFN and TNF (Brunner et al., 2011). These cytokines can contribute, along with the innate immune system, to the low‐grade pro‐inflammatory background observed in elderly individuals (Franceschi et al., 2006), which may worsen COVID‐19 outcomes and explain the elevated levels of inflammation. It is also possible that age‐associated clonal hematopoiesis may contribute to the increased inflammation due to hematopoietic stem cell myeloid generation bias of pro‐inflammatory macrophages and mast cells, and reduction of lymphoid differentiation (Jaiswal & Ebert, 2019). Moreover, decreased T‐cell capacity to properly activate antibody‐secreting cells to further elicit effective immune responses may be compromised (Haynes & Swain, 2006). Yet, another possible explanation is thymus involution (Palmer, Albergante, Blackburn, & Newman, 2018). During aging, the thymus becomes atrophic and is gradually replaced by fibrotic tissue (Flores, Li, Sempowski, Haynes, & Hale, 1999). This results in a reduced number, or even complete abrogation, of exiting naive T cells (Naylor et al., 2005). Together, all these features may result in the decreased ability of older people to fight viral infections, leading to age‐related inflammation and higher susceptibility of the lung, and eventually other organs, to the COVID‐19‐inflicted damage.

SARS‐CoV‐2 infects human host cells by attaching its membrane spike (S) protein to a receptor named angiotensin‐converting enzyme 2 (ACE2) (Letko, Marzi, & Munster, 2020). Activation of SARS‐CoV‐2 S protein is mainly mediated by the type II transmembrane serine protease (TMPRSS2), resulting in virus entry via endocytosis (Hoffmann et al., 2020). Therefore, the level of ACE2 expression may be a factor in SARS‐CoV‐2 infection. Single‐cell RNA‐sequencing studies have identified human cell subtypes vulnerable to SARS‐CoV‐2 infection by their co‐expression of ACE2 and TMPRSS2 in multiple tissues, importantly, in type II pneumocytes in the lung (Ziegler et al., 2020). ACE2 is a peptidase that converts angiotensin II to angiotensin 1‐7 (Vickers et al., 2002), attenuating its effects in vasoconstriction and inflammation. The expression levels of ACE2 are known to be affected by diverse stimuli and certain drugs used to treat hypertension (Ferrario et al., 2005), diabetes, and other diseases. How ACE2 expression is regulated by aging has been addressed recently, reporting no significant increase with age (Chen et al., 2020; Li, Li, Zhang, & Wang, 2020). Increased ACE2 mRNA levels, however, were observed in nasal epithelium in adults compared to children (Bunyavanich, Do, & Vicencio, 2020).

In this work, we revealed a strong link between COVID‐19 fatality rate and aging. Based on our analysis, we propose that COVID‐19, and more generally deadly respiratory diseases, should be considered as novel and emergent diseases of aging. Understanding that age is a major factor for fatality of COVID‐19 may help to design approaches against this disease that target the aging process, along with specific antiviral approaches and those that boost more efficiently the human immune system of the elderly.

## 2 RESULTS

**FIGURE 1:**
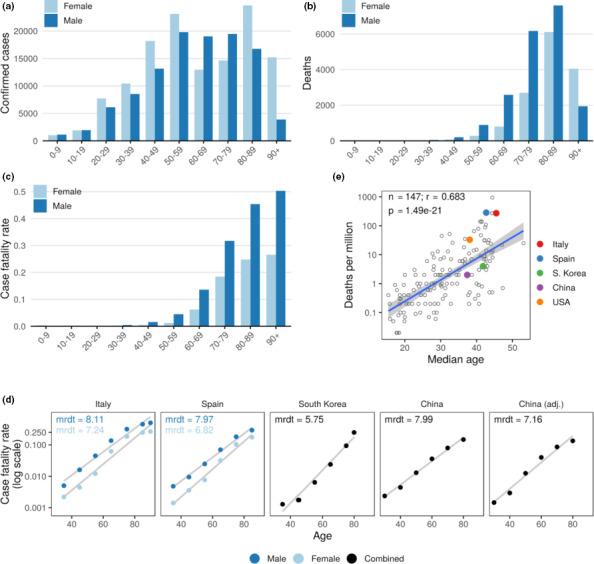
Age is a major risk factor for COVID‐19. (a) Confirmed cases of COVID‐19 in Italy. Data are through June 23, 2020. (b) Deaths in Italy. Men are in dark blue, and women in light blue. (c) Case fatality rate in men and women across age groups. (d) Mortality rate exponentially increases with age. Case fatality rate as a function of age for indicated countries. Linear regression of log of case fatality rate on age is shown by light lines. Men are shown in dark blue and women in light blue. Combined cases (men + women) are shown in black for indicated countries. mrdt—mortality rate doubling time. (e) COVID‐19 mortality as a function of the median age of the country. The countries discussed in the study are labeled

We initially analyzed epidemiological data on SARS‐CoV‐2 infections in Italy as of June 23, 2020 (“ISS”, 2020). In total, 239,709 cases were reported positive for SARS‐CoV‐2, with the median age of patients being 61 years. 70% of the infected individuals were 40 years or older (Figure 1a); however, there was no consistent trend toward an increased incidence rate in older people. 33,542 patients had reportedly died, with 85% of the fatalities observed in subjects 60 years old or older (Figure 1b). Women were more prevalent among confirmed cases (54.2%; 95% confidence interval (CI) 54.0–54.4), whereas men were more prevalent among deceased patients (58.1%; 95% CI 57.5–58.6).

The case fatality rate (CFR) is commonly used to assess disease severity and to estimate the outcome of a disease. We found that the CFR of COVID‐19 exponentially grows with age in both genders, exceeding 45% in people above 80 years old (Figure 1c), whereas it was low in young adults and children (Figures S1 and S2). This pattern is reminiscent of all‐cause mortality in the human population, which doubles approximately every 8 years, also known as mortality rate doubling time (MRDT). Based on the COVID‐19 CFR data, we assessed the doubling time of COVID‐19 deaths using the Gompertz law (Figure 1d). The doubling time was 8.1 years for men and 7.2 years for women. We then extended the analyses to other countries and found that the doubling times ranged from 5.75 to 7.99 years for Spain, China, and South Korea (Figure 1d).

Estimation of CFR of an infectious disease during an outbreak is challenging. First, there is a time lapse between a person developing the symptoms, the case being detected and reported, and the outcome of the disease. Second, the detection of a newly emerged pathogen is biased toward clinically severe cases. Therefore, naive CFR (ratio of fatalities over confirmed cases) can be inaccurate in the assessment of disease severity, which could explain differences observed between different countries. Adjusted CFR attempting to correct for these effects have been produced by several groups (Verity et al., 2020; Wu, Zhao, et al., 2020). Here, we used the CFR estimates adjusted for underlying demography and potential under‐ascertainment of mainland China (Verity et al., 2020). This allowed us to compare the doubling time of naive CFR and adjusted CFR. The doubling time obtained using the adjusted CFR was 7.16 years, approximately 10% shorter than the naive CFR. Nonetheless, the doubling time using adjusted CFR was not substantially different compared to other countries (Figure 1d). We further found that the rate of death from COVID‐19 (adjusted to population size) grows exponentially with the median age of the country (Figure 1e), presumably because countries with older populations have a higher fraction of people who may succumb to this disease. The incidence of COVID‐19 also grows exponentially with the median age of the country (Figure S3). Overall, the data show that not only CFR for COVID‐19 grows exponentially with age and that its doubling time approaches that of all‐cause human mortality. Together with the higher mortality for men than for women, this finding establishes COVID‐19 as a disease of aging, with age emerging as a major risk factor for COVID‐19.

**FIGURE 2:**
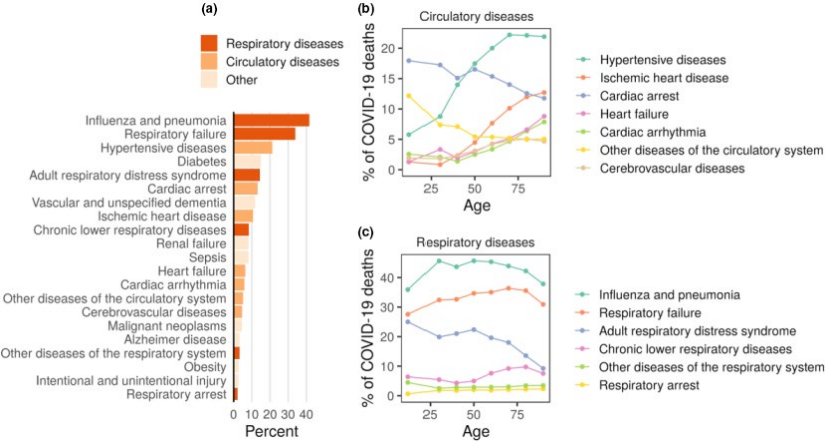
Conditions contributing to deaths involving COVID‐19. (a) Percentage of deceased patients diagnosed with the corresponding comorbidity in the United States as of June 22, 2020. (b–c) Age‐related percentages for (b) circulatory diseases and (c) respiratory diseases. Other diseases are shown in Figure S5

People with certain underlying medical conditions are thought to be at an increased risk of severe illness from COVID‐19. We explored age as a factor for the presence of health conditions contributing to COVID‐19 deaths. A total of 106,008 deaths involving COVID‐19 were reported in the United States as of June 22, 2020 (“CDC”, 2020). For 7% of the deaths, COVID‐19 was the only cause, and for deaths with conditions in addition to COVID‐19, on average, there were 2.5 additional conditions or causes of death. The most common conditions were pneumonia and influenza (42% of deaths), respiratory failure (34%), hypertension (21%), and diabetes (15%) (Figure 2A). Similar percentages were also observed in Spain and Italy (Figure S4). We explored the relationship between the presence of comorbidities in COVID‐19 deaths and age through correlation analysis (Table S1). We observed an age‐related increase in the proportion of patients for several circulatory diseases; notably, the presence of hypertensive diseases increased from 5% in the youngest age group to more than 22% in patients above 60 years old (Figure 2B). However, hypertension is strongly associated with age, and therefore, it is difficult to disentangle the effects of each. Similarly, Alzheimer's disease and dementia were also increased with age (Figure S5). On the other hand, the proportion of patients with respiratory diseases, including pneumonia, appeared to be mostly flat across age groups (Figure 2c). This suggests that the increased risk of death from COVID‐19 in elderly individuals cannot be ascribed to an increased risk of pneumonia. In order to dissociate the risk imposed by age‐related diseases and aging, we analyzed the mortality rate in deceased patients who had no additional health conditions in their death certificate (Table S2). The mortality rate in those patients also increased exponentially with age (Figure S6), suggesting that the increased risk of dying from COVID‐19 observed in elderly subjects is not only due to age‐associated diseases, but due to aging itself.

**FIGURE 3:**
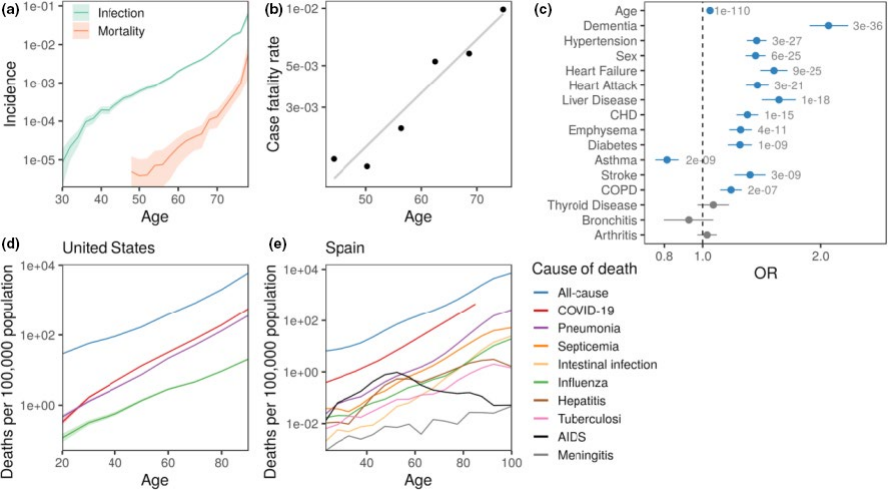
Pneumonia as an aging‐related disease. (a) Incidence of pneumonia (green) and deaths with pneumonia as the primary cause (red) across age groups in the UKB cohort. (b) Case fatality rate as a function of age in UKB. Linear regression of log of case fatality rate on age is shown by a light line. (c) Risk factors associated with death from pneumonia. Logistic regression odds ratio (OR) for UKB phenotypes. Phenotypes with *p*‐value < 0.05 marked in blue. (d) Mortality rate of COVID‐19 (red), pneumonia (purple), influenza (green), and all‐cause mortality (blue), in the United States from Feb 1, 2020, to June 27, 2020. (e) Mortality rate of pneumonia, COVID‐19, and other infectious diseases in Spain. COVID‐19 deaths recorded from Feb 11, 2020, to May 10, 2020 (89 days). The mortality rate for the rest of causes corresponds to the 5‐year average (2014 to 2018) normalized to a period of 89 days. All panels combine men + women

COVID‐19 is not the only disease caused by pathogens and leading to severe and often deadly pneumonia. To get a broader view on the relationship between aging and lung disease, we analyzed the UK Biobank (UKB) dataset and observed that the incidence of pneumonia (the UKB disease code J18, “pneumonia, the organism is unspecified”) was also higher in men than in women and increased exponentially with age (Figure 3a). The disease incidence doubling time was approximately 5 years for both sexes.

The disease cases covered under the J18 label in the UKB were recorded by the hospitals. Overall, among 410,351 medical records, 10,109 participants had at least one episode of pneumonia recorded (18,493 in total), and in 104 patients, pneumonia was listed as the primary cause of death. The pneumonia CFR grows exponentially for patients exceeding the age of 50 (Figure 3b). The CFR doubling time was 9 years (with a large 95% CI from 5.6 to 12.4 years due to a low number of terminal cases), which is again compatible with both all‐cause MRDT and COVID‐19 CFR doubling time. The prevalence of age‐related diseases was also a risk factor determining the probability of death from pneumonia. This could be seen from a series of univariate logistic models to determine the effects of age, sex, and prevalence of pre‐existing conditions on the patient survival (Figure 3c). The log‐odds ratio (log‐OR) associated with age was 0.04 (*p* < 1e‐110), which is equivalent to the CFR doubling time of approximately 10 years that is exactly the CFR doubling time in Figure 3b. Thus, like COVID‐19, pneumonia is a disease of aging. Pneumonia also showed increasing mortality rates with age in the United States, though their rates were lower than COVID‐19 in adults (Figure 3d). Other infectious diseases also exhibited elevated mortality rates with age, with the notable exceptions of AIDS and meningitis (Figure 3e).

Several studies report on the role of ACE2 as the SARS‐CoV and SARS‐CoV‐2 transmembrane receptor (Hoffmann et al., 2020; Li, Li, Farzan, & Harrison, 2005). We hypothesized that age‐dependent changes in the expression of ACE2 may contribute to the severity of the disease or to mortality of the elderly. A recent study (not peer reviewed) analyzed ACE2 expression levels in various datasets with regard to confounding factors such as sex, smoking, age, and race (Cai, 2020). Although it did not find an effect of aging, it is possible it may be due to insufficient sample size. Therefore, we examined human RNA‐seq samples from the GTEx project (https://gtexp​ortal.org), the largest gene expression dataset of subjects across the adult lifespan, to assess the expression of ACE2 across multiple tissues.

We analyzed the relationship between ACE2 expression and age across human tissues. Because GTEx comprises a heterogeneous set of samples, we sought to correct by the following covariates: sex, race, BMI, hypertension, and the use of a ventilator at the time of death. We implemented a linear mixed model including those five covariates as random effects (Methods). In five tissues out of the 44 analyzed (after excluding sex‐specific tissues and those with too few samples), we observed a moderate positive correlation between ACE2 expression and age after *p*‐value correction (FDR< 0.05): esophagus gastroesophageal junction, esophagus muscularis, lung, skeletal muscle, and liver (Table S3). The fitted regression models showed that the use of a ventilator at the time of death has the strongest effect (high random effect variance), more so than race, BMI, sex, and hypertension (Figure S7). Indeed, ACE2 expression in samples from subjects on a ventilator at the time of death is higher in many tissues (Figure S8).

**FIGURE 4:**
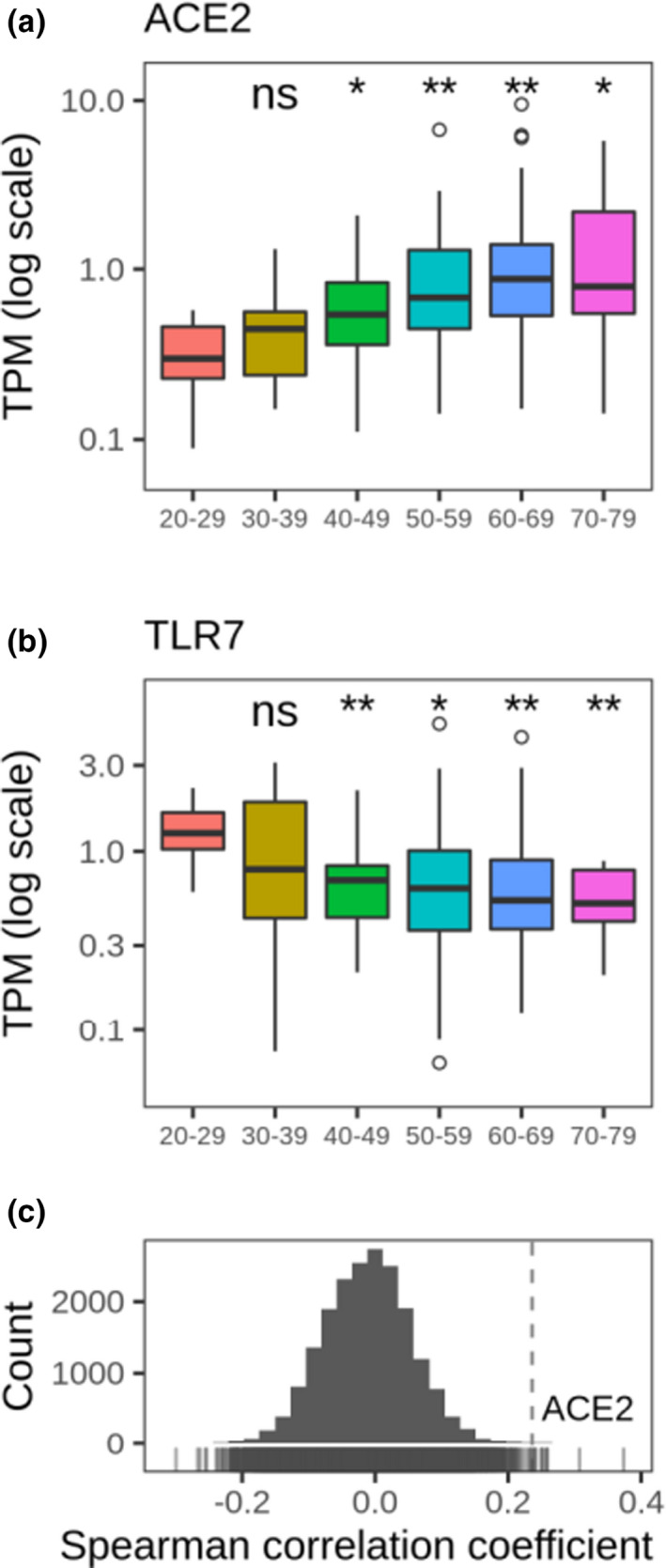
Age‐related changes in SARS‐CoV‐2 receptor (ACE2) gene expression. Expression of (a) ACE2 and (b) TLR7 mRNAs in human lung samples. TPM (Transcripts Per Million) corresponds to normalized RNA‐seq expression. *p*‐values were calculated using the Mann‐Whitney test; ns—not significant, *p < 0.05, **p < 0.01. (c) Distribution of genome‐wide correlations between age and expression of genes in the lung. ACE2 is one of top genes whose expression increases with age. GTEx was used for these analyses. Cases on ventilator immediately before death were excluded

The lung is one of the organs considered vulnerable to the SARS‐CoV‐2 infection based on the expression of ACE2 and TMPRSS2 in type II pneumocytes (Qi, Qian, Zhang, & Zhang, 2020; Ziegler et al., 2020; Zou et al., 2020). The expression of ACE2 in the lung increases with age in subjects who were not on a ventilator at the time of death (Pearson's *r* = 0.23; *p* = 0.0002; *n* = 261) (Figure 4a). This observation was consistent in both men and women (Figure S9). We further analyzed age‐related changes in gene expression for all genes expressed in the lung. ACE2 exhibited one of the highest correlation coefficients with age in patients that were not on a ventilator (Figure 4c). It is of interest that ACE2 is also upregulated by several drugs that are used to treat hypertension, a dominant pre‐existing condition associated with COVID‐19 mortality (Figure 2).

TMPRSS2 is known to be an ACE2 partner protein that facilitates the virus entry (Hoffmann et al., 2020). We assessed the relationship between TMPRSS2 expression and age, but did not find any tissue in which TMPRSS2 increased expression with age, reaching significance level (Table S4). Co‐expression of ACE2 and TMPRSS2 genes was also assessed across tissues in GTEx samples. We observed the strongest correlation of expression between the two genes in the small intestine and colon. The lung showed a much higher expression of TMPRSS2 than ACE2, when compared to the median expression across tissues (Figure S10).

Furthermore, the expression of the Toll‐like receptor 7 (TLR7) gene, a major component of the antiviral immunity, decreases with age in the lung, which may contribute to a poor immune response in elderly patients (Figure 4b). The expression of ACE2 as a function of age was analyzed in GTEx samples from subjects diagnosed with hypertension and other common COVID‐19 comorbidities (Figures S11 and S12). In addition, we evaluated the expression of genes related to the immune function in the lung and observed gender‐associated differences, for example, an age‐related upregulation of pathways, such as innate immune response and negative regulation of adaptive immunity. Interestingly, the viral genome replication pathway was found to be upregulated (Figure S13).

Studies in rat lungs showed a decrease in ACE2 expression with age (Xudong, Junzhu, Xingxiang, Furong, & Yanrong, 2006). While further analyses are needed, our study suggests that the opposite might be true in humans. Increased expression with age of ACE2 mRNA was observed in the nasal epithelium in children vs. adults (Bunyavanich et al., 2020). Age‐associated changes in ACE2 expression at the entry point of the virus into the cells may contribute to the age‐related increase in the SARS‐CoV‐2 infections and mortality.

## 3 DISCUSSION

Our study establishes COVID‐19 as an emergent disease of aging. This conclusion is based on (a) an exponential growth of its CFR with age, (b) the COVID‐19 mortality rate doubling time approaching that of all‐cause human mortality, (c) higher mortality in men than in women, (d) strong association with pre‐existing age‐related diseases, (e) COVID‐19 being a subset of all‐cause pneumonia, which is itself a disease of aging, and (f) an age‐related increase in the SARS‐CoV‐2 receptor mRNA expression in the lungs of non‐ventilator subjects. Together, this puts COVID‐19 in line with the archetypical diseases of aging, such as type II diabetes, cancer, Alzheimer's disease, and cardiovascular diseases.

The SARS‐CoV‐2 infection rate also showed an age‐related increase (Figure 1a and Figure S2); however, this was primarily due to a low incidence among children and young adults, whereas its subsequent age‐related changes were inconsistent. Moreover, testing priorities, age structure of the population, and reporting procedures may bias this analysis (e.g., younger people may be asymptomatic and therefore tested less). Further studies are needed to assess the effect of age on the infection rate. Like incidence, CFR substantially varies among the countries (e.g., it is 10‐fold higher in Italy and Spain than in Germany), which may also be explained by different strategies of reporting and testing, or differences in the healthcare infrastructure. This, however, should not affect the age‐related patterns in our study as we compare age groups within countries and not across them. Indeed, we observe similar COVID‐19 mortality rate doubling times across all countries examined, even though they report drastically different incidences, testing and reporting strategies, and CFRs.

Age‐adjusted all‐cause mortality is known to be higher in men than in women, and, as a result, women live longer than men. In the case of COVID‐19, this trend seems to be even stronger. For example, across most age groups, men are twice more likely to die from COVID‐19 than women in Italy (Figure 1c). However, the population age structure may explain the higher mortality in women older than 90 years. Pre‐existing conditions substantially increase the risk of death from COVID‐19. These are primarily age‐related diseases, that is, hypertension, type II diabetes, and cardiovascular diseases. This risk is even higher in patients with multiple comorbidities. Overall, it further supports the notion that COVID‐19 is an age‐related disease.

Many COVID‐19 patients die from severe pneumonia triggered by the specific virus infection. Other pathogens are also known to cause pneumonia, which is particularly deadly for the elderly; however, older people are not always affected by viral infection or pneumonia, for example, the largest pandemic in medical history—the Spanish influenza—affected young people (<65 years old) disproportionately more severely, with the peak mortality at around 30 years (Figure S14). Based on UKB clinical histories, we investigated pneumonia as a proxy for all complications from respiratory diseases. The pneumonia incidence increased exponentially with age, so that the disease incidence doubled every 5 years. This is close to the all‐cause MRDT from the Gompertz mortality law. Our observation of increasing incidence rate with age is consistent with previous studies of the incidence and MRDT of community acquired pneumonia in Germany (Ewig et al., 2009). The mortality associated with pneumonia increased faster in the elderly patients. The corresponding CFR increased exponentially with age for patients older than 50, and the CFR doubling time, again, was close to the Gompertz exponent. As in the case with COVID‐19, risks of death from pneumonia were significantly higher in men and in patients with pre‐existing conditions, such as hypertension, diabetes, and coronary heart disease, but not in patients with, for example,bronchitis. A similar pattern was also seen in a recent study of the clinical course of COVID‐19 (Zhou et al., 2020). The common risk factors of severe pneumonia caused by COVID‐19 and by infections in UKB suggest shared and pathogen‐independent mechanisms. The appearance of the incidence and CFR acceleration rates close to the all‐cause MRDT from the Gompertz mortality law is a signature of the first‐order effects of aging. Together, this links COVID‐19 and pneumonia as diseases of aging.

The association of COVID‐19 CFR with aging as well as the association of the incidence and CFR of pneumonia with aging may open a way for using future anti‐aging drugs, such as senolytics (Ogrodnik et al., 2017; Xu et al., 2018), for both the prophylaxis and treatment of potentially deadly infectious diseases. First treatments seem to reduce markers of frailty and inflammation in pre‐ and clinical trials (Kirkland, Tchkonia, Zhu, Niedernhofer, & Robbins, 2017) and hence may be helpful to prevent deadly complications. State‐of‐the‐art biomarkers of aging, such as DNA methylation‐based clocks (Horvath & Raj, 2018), could be used to stratify patients to define cohorts for expedite clinical trials and subsequently to select the most vulnerable individuals for treatment.

The mechanistic basis for the age‐related pattern of COVID‐19 mortality remains unclear. Our finding of an elevated age‐related expression of ACE2 in the lungs of subjects with non‐ventilator deaths may provide a clue. ACE2 is the site for the entry of SARS‐CoV‐2 into the cell. An age‐related increase in the expression of this gene, together with the depletion of antiviral defenses, would naturally support a higher damaging effect of the coronavirus in the lung. It should be noted, however, that while ACE2 specifically promotes SARS‐coronavirus infections, it also protects lungs from injury (Monteil et al., 2020). In addition, various tissues harbor different ACE2 gene expression levels and may account for complications other than pneumonia, such as diarrhea observed in COVID‐19‐positive patients. At the protein level, lung and its alveolar type II cells were found to have low or undetectable ACE2 protein levels. Although we observed a moderate increased ACE2 mRNA levels with age in the lung, one limitation of using bulk RNA‐seq is that we could not differentiate between increased expression in a particular cell type, or overrepresentation of ACE2+ cell populations. Interestingly, ventilator cases showed no increase in ACE2 expression with age. The main difference between ventilator and non‐ventilator cases is in the young subjects, wherein the expression of ACE2 in the ventilator cases is higher (Figure S15). The implications of variable expression patterns of ACE2 mRNA and protein across ages, tissues, and ventilator cases should be investigated in further studies.

Many therapeutic strategies, such as those that support sequestration of the virus and protect lungs by a soluble form of ACE2, are currently under development (Vu, Farish, Jenkins, & Kelly, 2002). These and other strategies may be useful as the SARS‐CoV‐2 vaccine is not yet available. Moreover, vaccines are less efficient in the elderly, leading to a high rate of infections even in vaccinated individuals. For example, the yearly influenza vaccine is only 40%–60% efficient in older individuals (Vu et al., 2002). Therefore, targeting viral (and the associated host) pathways may be viewed as both an immediate and a long‐term strategy. However, as the case fatality rate grows with age, it should be possible to also adjust the biological age thereby targeting COVID‐19. There are plenty of candidates for such a strategy (Zhavoronkov, 2020).

Overall, our analysis indicates that COVID‐19 exhibits a clear characteristic of an age‐related disease and that age and age‐related diseases are its major risk factors. Currently, there is a strong push toward therapeutic approaches targeting the machinery involved in viral biology. Our research suggests that targeting the aging process itself can be a viable orthogonal strategy against COVID‐19 and other deadly respiratory diseases.

## 4 METHODS

### 4.1 Epidemiological analyses of COVID‐19

We obtained epidemiological data for Italy from *Istituto Superiore di Sanità*, as of June 23, 2020 (“ISS”, 2020), for Spain from *Instituto de Salud Carlos III*, as of May 10, 2020 (“ISCIII”, 2020), for South Korea from Korea Centers for Disease Control & Prevention, as of July 4, 2020 (“KCDC”, 2020), and for mainland China from January 1, 2020, to February 11, 2020, from (Verity et al., 2020). We used the reported number of confirmed cases and deaths to compute the age‐stratified case fatality rate. For Italy and Spain, the data were also broken down by sex to compute gender‐specific case fatality rate. COVID‐19 is currently a developing pandemic, which poses important challenges when assessing its CFR. We also used the adjusted case fatality rate, which might provide a more accurate estimate of the severity of COVID‐19 (Verity et al., 2020). The model in which the estimate was obtained assumes that attack rates (i.e., the probability of becoming infected) do not vary substantially by age and takes into account the underlying demography and potential under‐ascertainment (Verity et al., 2020). This allowed us to compare the doubling time obtained using the naive CFR (ratio of reported deaths over confirmed cases) with the one obtained using the adjusted CFR.

We used the number of COVID‐19 deaths by age reported by the National Center for Health Statistics of the CDC (“NCHS”, 2020) between February 1 and June 27, 2020, to compute the mortality rates reported in Figure 3d. The mortality rates for pneumonia in Figure 3d were computed by subtracting the “Pneumonia and COVID‐19 Deaths” from the “Pneumonia Deaths,” as the latter includes deaths with or without COVID‐19. The number of deaths from infectious diseases in Spain by age shown in Figure 3e, and the Spain population to compute the mortality rates were downloaded from Instituto Nacional de Estadistica (https://www.ine.es/index.htm, last accessed July 13, 2020). The mortality rates were computed as the 5‐year average between 2014 and 2018, normalized to a period 89 days, in order to compare with the mortality rate of COVID‐19, which corresponded to the period from February 11, to May 10, 2020 (89 days).

### 4.2 Gene expression data from GTEx

Data on gene expression were obtained from the Genotype‐Tissue Expression (GTEx) project (dbGaP accession number phs000424.v8.p2). GTEx provides bulk mRNA sequencing (RNA‐seq) from a large cohort of post‐mortem, organ donor, and surgical donor samples across 54 tissues and 948 individuals. A total of 17,382 samples were used in this work. We used normalized gene expression qualifications (TPM, Transcripts Per Million) to compute the correlation between age and ACE2 gene expression. The covariate DTHVNT (Donor On A Ventilator Immediately Prior To Death; dbGaP accession phv00169090.v4.p1) has a strong effect on the expression of ACE2 in the lung. This subject phenotype is described as “Assertion that donor was assisted by a medical device that facilitates breathing immediately prior to death.” The number of subjects, whose lung samples are available, are distributed as follows regarding the use of a ventilator: yes = 261; no = 313; unknown = 3. Figure S15 shows the difference in ACE2 expression in the lung between ventilator and non‐ventilator samples across age groups. The difference in expression of ACE2 between ventilator and non‐ventilator samples across different tissues is shown in Figure S8. The gender‐specific expression of ACE2 in the lung is shown in Figure S9. We also analyzed phenotypes related to common comorbidities in COVID‐19. The subject phenotypes were hypertension (MHHTN), ischemic heart disease (MHHRTDIS), chronic respiratory disease (MHCOPD), renal failure (MHRNLFLR), cerebrovascular disease (MHCVD), type 1 diabetes (MHT1D), type 2 diabetes (MHT2D), and history of non‐metastatic cancer (MHCANCERNM). The expression of ACE2 as a function of age in those phenotypes is shown in Figure S11. The gene TPM values were obtained from the file GTEx_Analysis_2017‐06‐05_v8_RNASeQCv1.1.9_gene_tpm.gct. The sample attributes were obtained from the file phs000424.v8.pht002743.v8.p2.c1.GTEx_Sample_Attributes.GRU.txt. And the subject phenotypes were obtained from the file phs000424.v8.pht002742.v8.p2.c1.GTEx_Subject_Phenotypes.GRU.txt.

### 4.3 Statistical analyses

Statistical analyses were performed using R (version 3.6.3). Linear mixed models (LMM) were implemented using the “lmer” function from the “lmertest” package. Variances of random effects were extracted using the “VarCorr” function from the “lme4” package. LMM were fitted using gene expression as log(TPM + 1), where TPM corresponds to Transcripts Per Million, and age, scaled using scale(AGE, scale = FALSE). LMM were fitted for ACE2 and TMPRSS2 using the following formula including random effects: lmer(log(TPM + 1) ~ AGE + (1|SEX) + (1|BMI) + (1|MHHTN) + (1|RACE) + (1|DTHVNT)); MHHTN corresponds to hypertension, and DTHVNT corresponds to the use of a ventilator at time of death. The correlation between age and gene expression for ACE2 was computed using the function “cor.test,” using AGE and TPM as parameters, to obtain Pearson's *r* or Spearman's rho, as indicated in the text. The genome‐wide correlations in non‐ventilator lung samples were computed using a custom Python script using the function “stats.spearmanr” from the “scipy” module. The mortality rate doubling time was computed as log(2)/p1, where p1 was obtained by fitting a linear regression model (p1*Age + p2) using “lm(log(CFR) ~ AGE)”, where CFR corresponds to case fatality ratio (deaths/confirmed cases) across ages. All figures were generated using the package ggplot2 (version 3.3.0).

### 4.4 Gene set enrichment analysis

Ventilator cases were excluded from downstream analysis. MicroRNAs, pseudogenes, hypothetical proteins, and low‐expressed genes were also excluded. First, the differential expression was calculated for each age group versus young adults (20‐39 years old) for men and women separately using the limma package in R. Ranks were calculated as −log10(*p*‐value)*1 if logFC > 0, and −log10(*p*‐value)*(−1) if logFC < 0, where logFC is the fold change in gene expression between the corresponding age bin and young controls. Gene set enrichment analysis was performed using the *ClusterProfiler* package in R (Yu, Wang, Han, & He, 2012). We included terms that contain any of the terms: ‘vir’, ‘macrophage’, ‘neutrophil’, ‘inflam’, ‘immun’, ‘chemokine’, ‘cytok’, ‘T cell’, or ‘B cell’. Gene ontology terms were reduced using the *simplify()* function (modified to work with *gseGO* results) with a cutoff of 0.3. Normalized enrichment score values were plotted using *heatmap*.*2()* for terms that were significantly enriched (q‐value < 0.05).

### 4.5 Pneumonia case analysis

We used UK Biobank first occurrences data (category 1712) to define pneumonia comorbidities and hospital inpatient data (category 2000) to ascertain all pneumonia cases during lifetime. Death register data (category 100093) were used to estimate the lethality of pneumonia cases. Incidence rate plots were produced using custom Python scripts, and logistic regressions were performed by “glm” function from R.

## CONFLICT OF INTEREST

POF is a shareholder of Gero, and POF and AAZ are Gero employees.

## AUTHOR CONTRIBUTIONS

DS designed research studies, acquired data, analyzed data, and wrote the manuscript. JPC, AAZ, and AVS acquired data, analyzed data, and wrote the manuscript. MVG, BZ, CK, and SHY acquired and analyzed data. POF designed research studies and wrote the manuscript. VNG supervised the study, conceived the study, designed research studies, wrote the manuscript, and acquired funding.

## Data Availability

The data used for the analyses described in this manuscript were obtained from The Genotype‐Tissue Expression (GTEx) Project Portal (https://www.gtexp​ortal.org) and dbGaP accession number phs000424.v8.p2, and the UK Biobank Resource under Application Number 21988. Data citations: ISS, 2020, ISCIII, 2020 and KCDC, 2020.
