# Supplementary material for: COVID‐19 is an emergent disease of aging
Source: Aging Cell. 2020 Oct 1;19(10):e13230. doi: 10.1111/acel.13230 (PMC7576244; doi:10.1111/acel.13230)
Supplement: Supplementary file 1 [file ACEL-19-e13230-s001.docx]

**COVID-19 is an emergent disease of aging**

**Supplementary Materials**

1. Supplementary Tables
2. Supplementary Figures
3. Supplementary References

**Supplementary Tables**

**Supplementary Table 1.** Pearson’s *r* correlation between the proportion of COVID-19 deaths diagnosed with the corresponding condition and age. The proportion of deaths was computed across the age groups 12.5, 30, 40, 50, 60, 70, 80, and 90+. Data points are shown in Fig. 2 and Supplementary fig. 5.

| **Group** | **Condition** | **r** | **p-value** |
| --- | --- | --- | --- |
| Circulatory diseases | Cardiac arrest | -0.94 | 0.0005 |
|  | Cardiac arrhythmia | 0.86 | 0.0065 |
|  | Cerebrovascular diseases | 0.94 | 0.0006 |
|  | Heart failure | 0.92 | 0.0011 |
|  | Hypertensive diseases | 0.95 | 0.0003 |
|  | Ischemic heart disease | 0.96 | 0.0001 |
|  | Other diseases of the circulatory system | -0.85 | 0.0075 |
| Respiratory diseases | Adult respiratory distress syndrome | -0.89 | 0.0032 |
|  | Chronic lower respiratory diseases | 0.66 | 0.0756 |
|  | Influenza and pneumonia | 0.05 | 0.9037 |
|  | Other diseases of the respiratory system | -0.17 | 0.6805 |
|  | Respiratory arrest | 0.86 | 0.0062 |
|  | Respiratory failure | 0.56 | 0.1506 |
|  | Sepsis | -0.70 | 0.0542 |
|  | Unspecified dementia | 0.82 | 0.0122 |
|  | Diabetes | -0.12 | 0.7713 |
|  | Un/Intentional injury | -0.73 | 0.0382 |
|  | Malignant neoplasms | 0.20 | 0.6293 |
|  | Obesity | -0.95 | 0.0002 |
|  | Renal failure | 0.13 | 0.7671 |
|  | Alzheimer disease | 0.80 | 0.0181 |
|  | All other conditions and causes (residual) | -0.81 | 0.0159 |

**Supplementary Table 2.** Number of COVID-19 deaths in the U.S. reported by the CDC as of 24 July, 2020; number of COVID-19 deaths without additional health conditions in their death certificate, and the percentage over all COVID-19 deaths; total U.S. population. Data obtained from the CDC upon contacting them.

| **Age group** | **All COVID-19 deaths** | **No conditions** | **%** | **Population** |
| --- | --- | --- | --- | --- |
| 0-24 | 220 | 11 | 5.0 | 103856244 |
| 25-34 | 919 | 60 | 6.5 | 45697774 |
| 35-44 | 2341 | 188 | 8.0 | 41277888 |
| 45-54 | 6411 | 401 | 6.3 | 41631699 |
| 55-64 | 15554 | 938 | 6.0 | 42272636 |
| 65-74 | 26672 | 1518 | 5.7 | 30492316 |
| 75-84 | 33782 | 2055 | 6.1 | 15394374 |
| 85+ | 42041 | 2991 | 7.1 | 6544503 |

**Supplementary Table 3.** Regression coefficients (Estimate) obtained in the linear mixed models for log ACE2 expression ~ AGE, across GTEx tissues. Sex, race, BMI, hypertension, and the use of ventilator at time of death, were used as random effects.

| **Tissue** | **Estimate** | **error** | **pvalue** | **FDR** |
| --- | --- | --- | --- | --- |
| Esophagus - Gastroesophageal Junction | 0.0077 | 0.0021 | 0.0003 | 0.0134 |
| Esophagus - Muscularis | 0.0058 | 0.0018 | 0.0012 | 0.0155 |
| Lung | 0.0054 | 0.0017 | 0.0014 | 0.0155 |
| Muscle - Skeletal | 0.0036 | 0.0011 | 0.0018 | 0.0155 |
| Nerve - Tibial | -0.0047 | 0.0015 | 0.0014 | 0.0155 |
| Liver | 0.0064 | 0.0022 | 0.0039 | 0.0285 |
| Adrenal Gland | 0.0032 | 0.0013 | 0.0122 | 0.0767 |
| Artery - Tibial | 0.0021 | 0.0009 | 0.0177 | 0.0971 |
| Artery - Coronary | 0.0103 | 0.0044 | 0.0218 | 0.1039 |
| Colon - Transverse | -0.0083 | 0.0037 | 0.026 | 0.1039 |
| Whole Blood | -0.0003 | 0.0001 | 0.0255 | 0.1039 |
| Brain - Hypothalamus | 0.0018 | 0.0009 | 0.0504 | 0.1764 |
| Stomach | 0.0029 | 0.0015 | 0.0521 | 0.1764 |
| Thyroid | 0.0053 | 0.0029 | 0.0725 | 0.2279 |
| Breast - Mammary Tissue | 0.0059 | 0.0035 | 0.0974 | 0.2678 |
| Minor Salivary Gland | -0.0044 | 0.0026 | 0.0933 | 0.2678 |
| Heart - Atrial Appendage | 0.0035 | 0.0022 | 0.1143 | 0.2957 |
| Artery - Aorta | -0.0029 | 0.0022 | 0.1925 | 0.4706 |
| Brain - Caudate (basal ganglia) | 0.0009 | 0.0007 | 0.2097 | 0.4857 |
| Adipose - Visceral (Omentum) | 0.0037 | 0.0032 | 0.2552 | 0.5491 |
| Brain - Putamen (basal ganglia) | 0.0007 | 0.0006 | 0.2621 | 0.5491 |
| Brain - Hippocampus | 0.0011 | 0.001 | 0.2932 | 0.5863 |
| Adipose - Subcutaneous | 0.003 | 0.0033 | 0.3566 | 0.5899 |
| Brain - Cortex | 0.0005 | 0.0005 | 0.362 | 0.5899 |
| Brain - Nucleus accumbens (basal ganglia) | 0.0007 | 0.0007 | 0.3383 | 0.5899 |
| Brain - Substantia nigra | 0.0021 | 0.0021 | 0.323 | 0.5899 |
| Pancreas | -0.0019 | 0.002 | 0.3597 | 0.5899 |
| Spleen | 0.0002 | 0.0003 | 0.5095 | 0.8007 |
| Cells - Cultured fibroblasts | 0.0001 | 0.0002 | 0.5688 | 0.8202 |
| Cells - EBV-transformed lymphocytes | 0.0001 | 0.0001 | 0.5721 | 0.8202 |
| Skin - Not Sun Exposed (Suprapubic) | 0.0004 | 0.0007 | 0.5779 | 0.8202 |
| Brain - Anterior cingulate cortex (BA24) | 0.0004 | 0.0008 | 0.6315 | 0.8545 |
| Small Intestine - Terminal Ileum | 0.0047 | 0.01 | 0.6409 | 0.8545 |
| Brain - Spinal cord (cervical c-1) | 0.0005 | 0.0013 | 0.6693 | 0.8661 |
| Esophagus - Mucosa | -0.0004 | 0.0011 | 0.689 | 0.8661 |
| Brain - Cerebellar Hemisphere | 0.0002 | 0.0005 | 0.743 | 0.896 |
| Brain - Frontal Cortex (BA9) | 0.0002 | 0.0008 | 0.7942 | 0.896 |
| Kidney - Cortex | 0.0019 | 0.0069 | 0.7846 | 0.896 |
| Skin - Sun Exposed (Lower leg) | -0.0002 | 0.0008 | 0.7914 | 0.896 |
| Brain - Amygdala | -0.0001 | 0.0007 | 0.932 | 0.9862 |
| Brain - Cerebellum | 0 | 0.0004 | 0.9638 | 0.9862 |
| Heart - Left Ventricle | -0.0003 | 0.0028 | 0.9129 | 0.9862 |
| Pituitary | 0.0001 | 0.001 | 0.9578 | 0.9862 |
| Colon - Sigmoid | 0 | 0.0022 | 0.9996 | 0.9996 |

**Supplementary Table 4.** Regression coefficients (Estimate) obtained in the linear mixed models implemented using log TMPRRS2 expression ~ AGE, across GTEx tissues. Sex, race, BMI, hypertension, and the use of ventilator at time of death, were used as random effects.

| **Tissue** | **Estimate** | **error** | **pvalue** | **FDR** |
| --- | --- | --- | --- | --- |
| Colon - Transverse | -0.025097 | 0.007051 | 0.0006 | 0.0281 |
| Adipose - Subcutaneous | 0.002382 | 0.001292 | 0.0658 | 0.3648 |
| Brain - Cortex | -0.000673 | 0.000338 | 0.0478 | 0.3648 |
| Breast - Mammary Tissue | -0.00852 | 0.004624 | 0.0663 | 0.3648 |
| Cells - Cultured fibroblasts | -0.00058 | 0.000242 | 0.0169 | 0.3648 |
| Esophagus - Muscularis | -0.002639 | 0.001232 | 0.0487 | 0.3648 |
| Liver | 0.007513 | 0.003796 | 0.0502 | 0.3648 |
| Minor Salivary Gland | -0.006383 | 0.003306 | 0.0608 | 0.3648 |
| Brain - Hippocampus | -0.000768 | 0.000452 | 0.0935 | 0.4113 |
| Skin - Sun Exposed (Lower leg) | 0.003826 | 0.00223 | 0.0867 | 0.4113 |
| Brain - Nucleus accumbens (basal ganglia) | -0.000511 | 0.000329 | 0.1214 | 0.4855 |
| Esophagus - Gastroesophageal Junction | -0.002573 | 0.001747 | 0.1419 | 0.4904 |
| Lung | -0.003841 | 0.002631 | 0.1449 | 0.4904 |
| Brain - Caudate (basal ganglia) | 0.000342 | 0.00024 | 0.1567 | 0.4924 |
| Brain - Amygdala | -0.000967 | 0.000725 | 0.1892 | 0.5016 |
| Brain - Frontal Cortex (BA9) | 0.000316 | 0.000243 | 0.1938 | 0.5016 |
| Whole Blood | -0.000535 | 0.000396 | 0.177 | 0.5016 |
| Cells - EBV-transformed lymphocytes | -0.000299 | 0.00025 | 0.2345 | 0.543 |
| Esophagus - Mucosa | -0.003587 | 0.002971 | 0.2278 | 0.543 |
| Adipose - Visceral (Omentum) | 0.000725 | 0.000658 | 0.2711 | 0.5965 |
| Brain - Putamen (basal ganglia) | 0.000325 | 0.000313 | 0.3005 | 0.6296 |
| Brain - Substantia nigra | 0.000282 | 0.000281 | 0.3164 | 0.6329 |
| Colon - Sigmoid | -0.003867 | 0.004608 | 0.4021 | 0.7692 |
| Artery - Aorta | 0.000758 | 0.00111 | 0.4949 | 0.7761 |
| Artery - Coronary | -0.000395 | 0.000525 | 0.4517 | 0.7761 |
| Brain - Anterior cingulate cortex (BA24) | -0.000237 | 0.000428 | 0.5821 | 0.7761 |
| Brain - Cerebellum | -0.000506 | 0.000897 | 0.5732 | 0.7761 |
| Brain - Spinal cord (cervical c-1) | -0.00032 | 0.000488 | 0.5134 | 0.7761 |
| Heart - Atrial Appendage | 0.000278 | 0.000499 | 0.5779 | 0.7761 |
| Heart - Left Ventricle | 0.000175 | 0.000249 | 0.4833 | 0.7761 |
| Muscle - Skeletal | -0.000127 | 0.000193 | 0.5116 | 0.7761 |
| Stomach | 0.004996 | 0.007754 | 0.5231 | 0.7761 |
| Thyroid | 0.001812 | 0.002903 | 0.5342 | 0.7761 |
| Brain - Cerebellar Hemisphere | -0.000124 | 0.000354 | 0.726 | 0.9191 |
| Brain - Hypothalamus | 0.000146 | 0.000443 | 0.7423 | 0.9191 |
| Nerve - Tibial | -0.000137 | 0.000433 | 0.752 | 0.9191 |
| Pancreas | 0.000618 | 0.002158 | 0.7749 | 0.9216 |
| Artery - Tibial | 0.000081 | 0.000392 | 0.8363 | 0.9311 |
| Kidney - Cortex | -0.000757 | 0.004522 | 0.8675 | 0.9311 |
| Pituitary | -0.000305 | 0.002179 | 0.8887 | 0.9311 |
| Skin - Not Sun Exposed (Suprapubic) | 0.000637 | 0.003032 | 0.8339 | 0.9311 |
| Small Intestine - Terminal Ileum | -0.00131 | 0.008639 | 0.8798 | 0.9311 |
| Adrenal Gland | 0.000008 | 0.000288 | 0.9778 | 0.9976 |
| Spleen | 0.000002 | 0.000597 | 0.9976 | 0.9976 |

**Supplementary Figures**


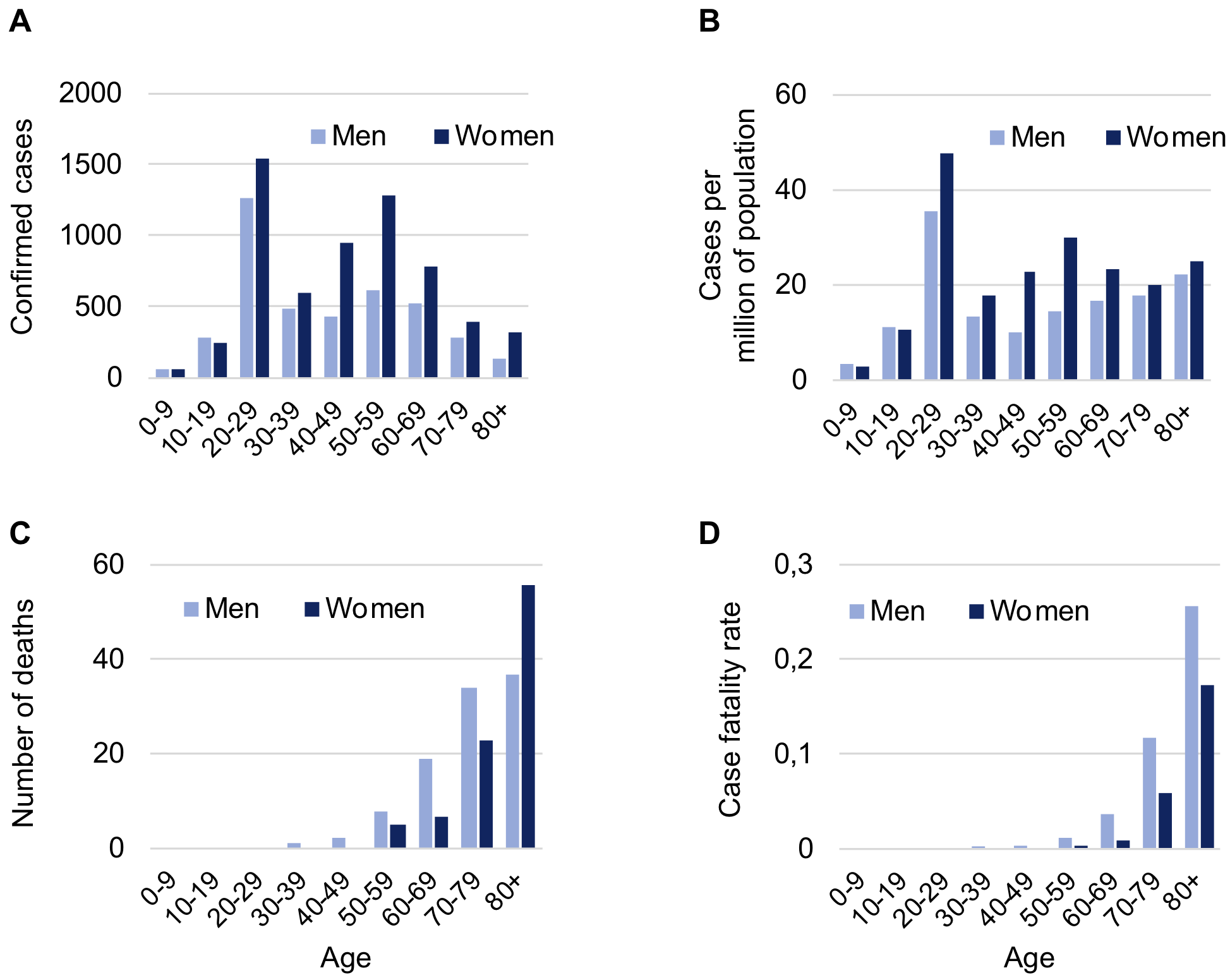


**Supplementary figure 1.** Confirmed COVID-19 cases and deaths across age groups and genders in South Korea. (A) Confirmed cases. (B) Confirmed cases per million of population. (C) Number of deaths. (D) Case fatality rate. Men are in light blue, and women in dark blue. Data are for Korea through April 7, 2020. Source (3).


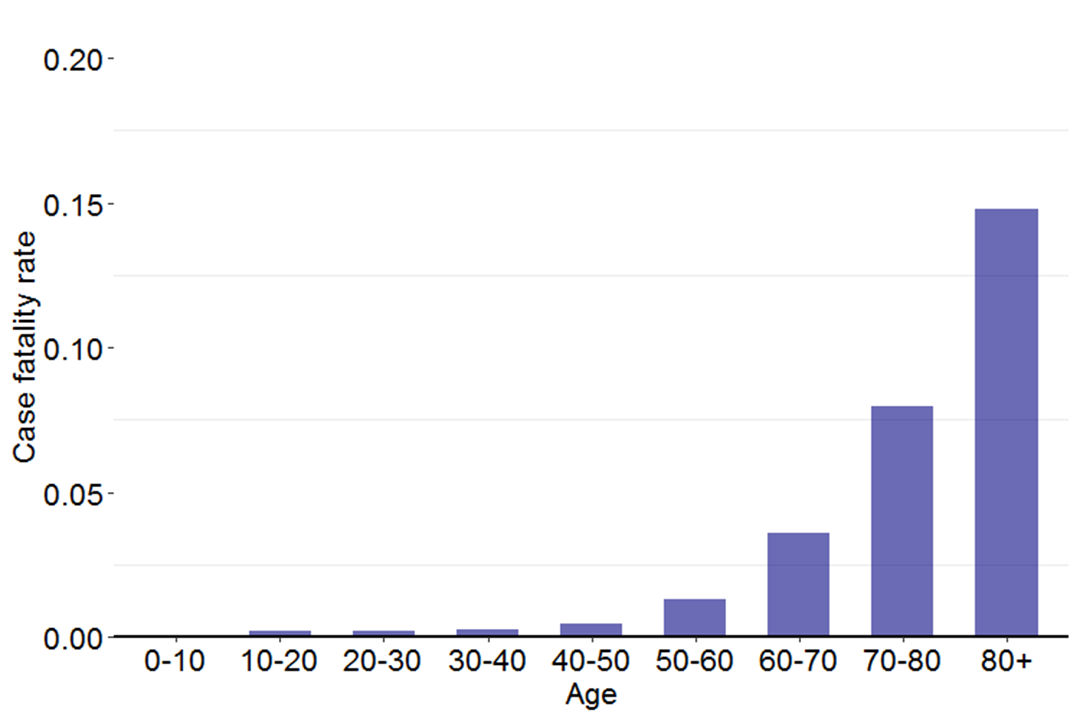


**Supplementary figure 2**. Case fatality rate in China across age groups, up to Feb 7, 2020. Source (5).


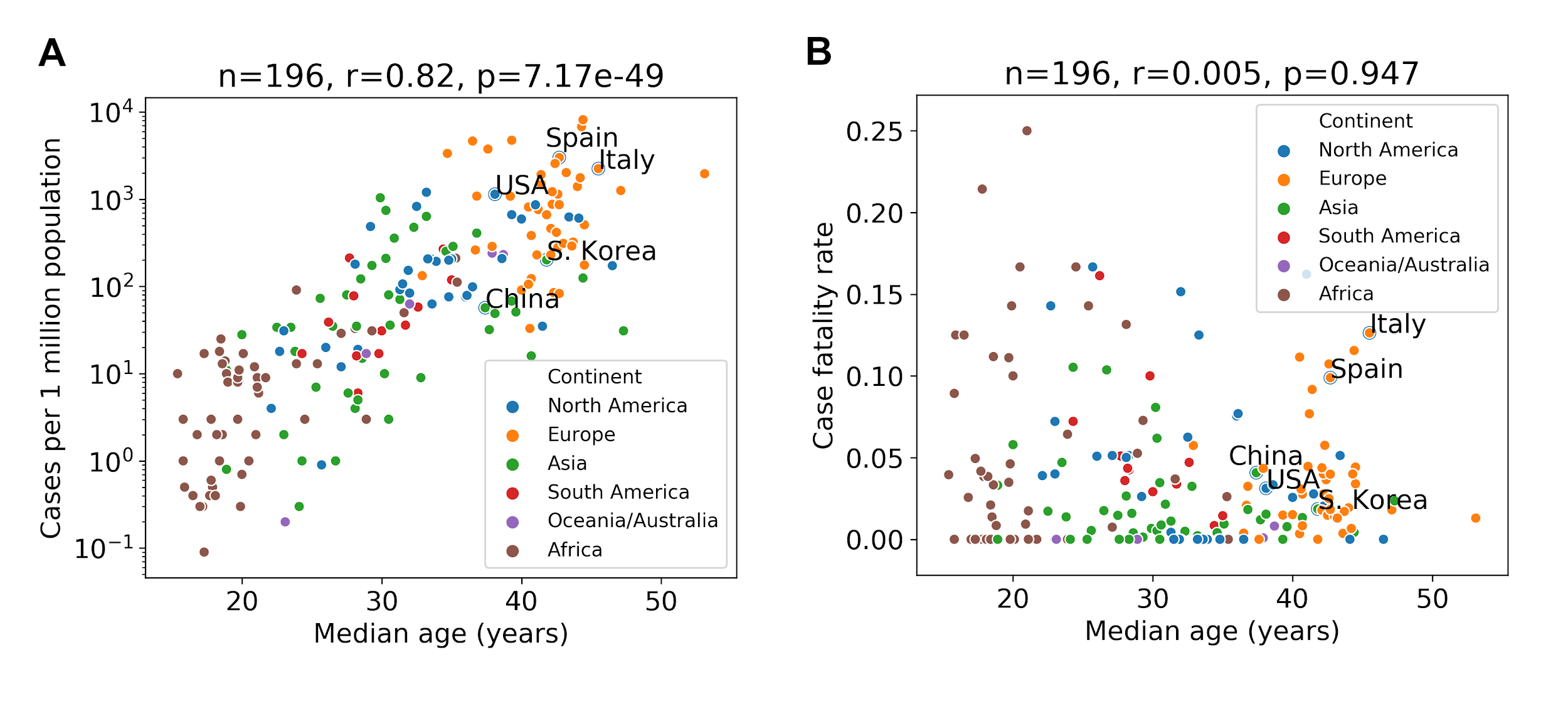


**Supplementary figure 3**. COVID-19 incidence across countries. (A) Incidence of COVID-19 as a function of the median age of the country. (B) Case fatality rate as a function of the median age of the country.

**
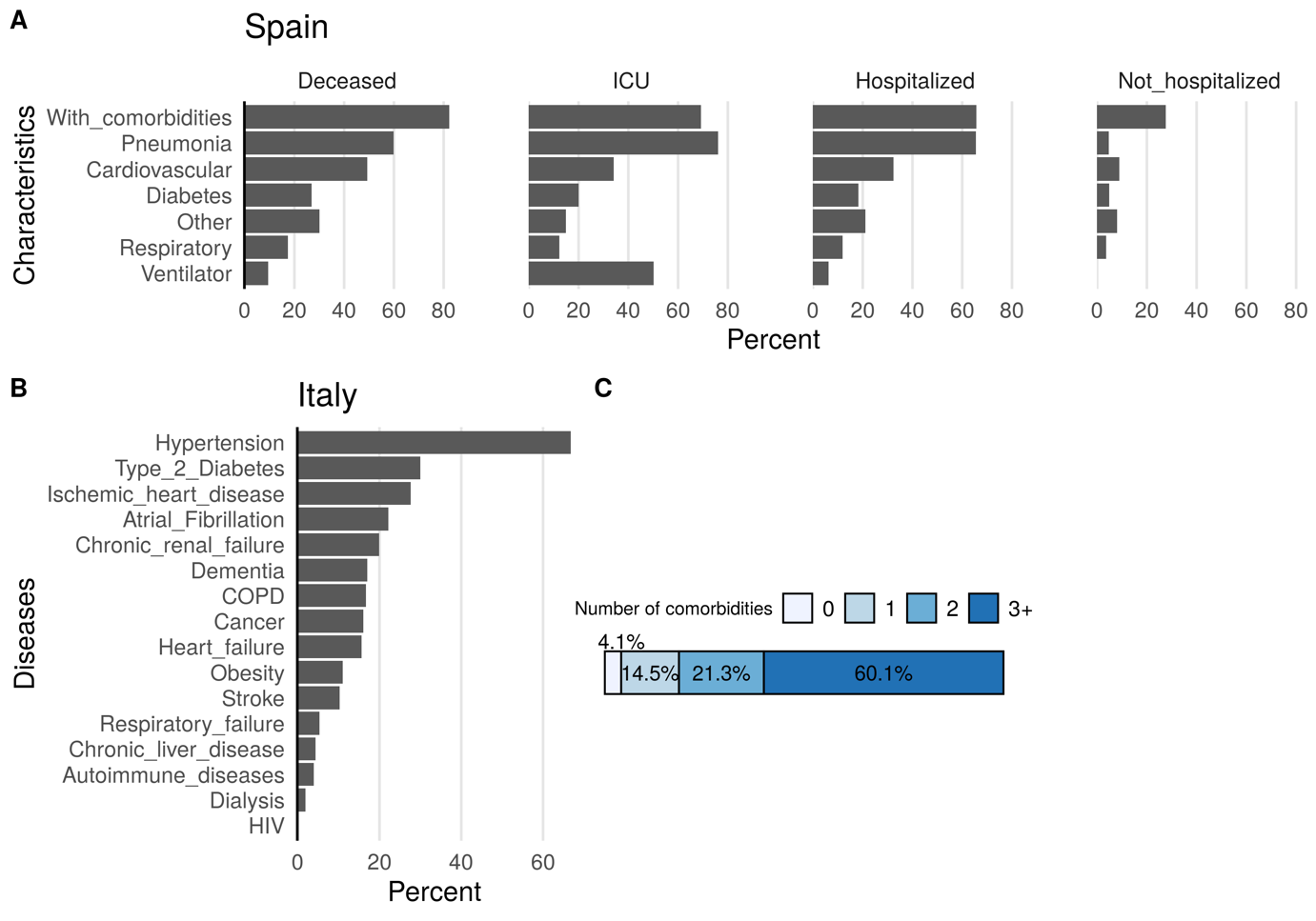
Supplementary figure 4**. Comorbidities and characteristics of COVID-19 patients in Spain and Italy. (A) Proportion of COVID-19 confirmed cases with the corresponding characteristics in Spain by severity of illness, as of 10 May 2020. Totals: deaths 20,534; ICU 7,695; Hospitalized 92,113; Not hospitalized 147,702. Source: Equipo COVID-19, RENAVE, CNE, CNM (ISCIII). ICU: Intensive Care Unit. (B) Common comorbidities observed in deceased patients in Italy, diagnosed before SARS-CoV-2 infection, as of 25 June 2020. (C) Percent of deceased patients with zero, one, two, or three or more comorbidities. Total patients: 3,602. Source: Epicentro, Istituto Superiore di Sanità. COPD: Chronic Obstructive Pulmonary Disease; Cancer: Active cancer in the past 5 years.

**
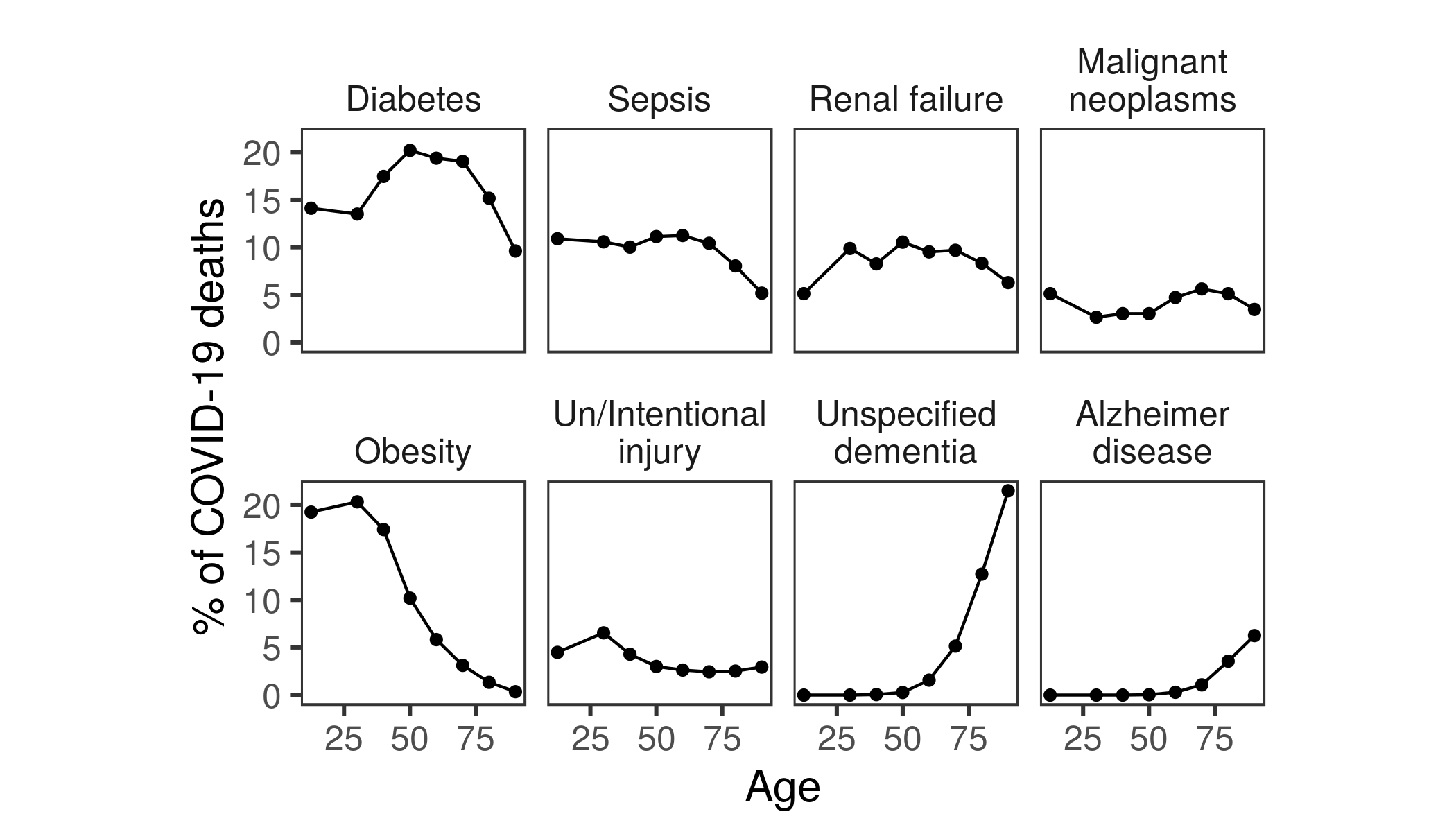
Supplementary figure 5**. Conditions contributing to deaths involving COVID-19 in the United States as of 22 June 2020, broken down by age groups. The proportion of patients diagnosed with the corresponding condition in each age group is shown. Total deaths: 106,008. Source: NCHS CDC. Un/intentional injury: Intentional and unintentional injury, poisoning and other adverse events; Unspecified dementia: Vascular and unspecified dementia.

**
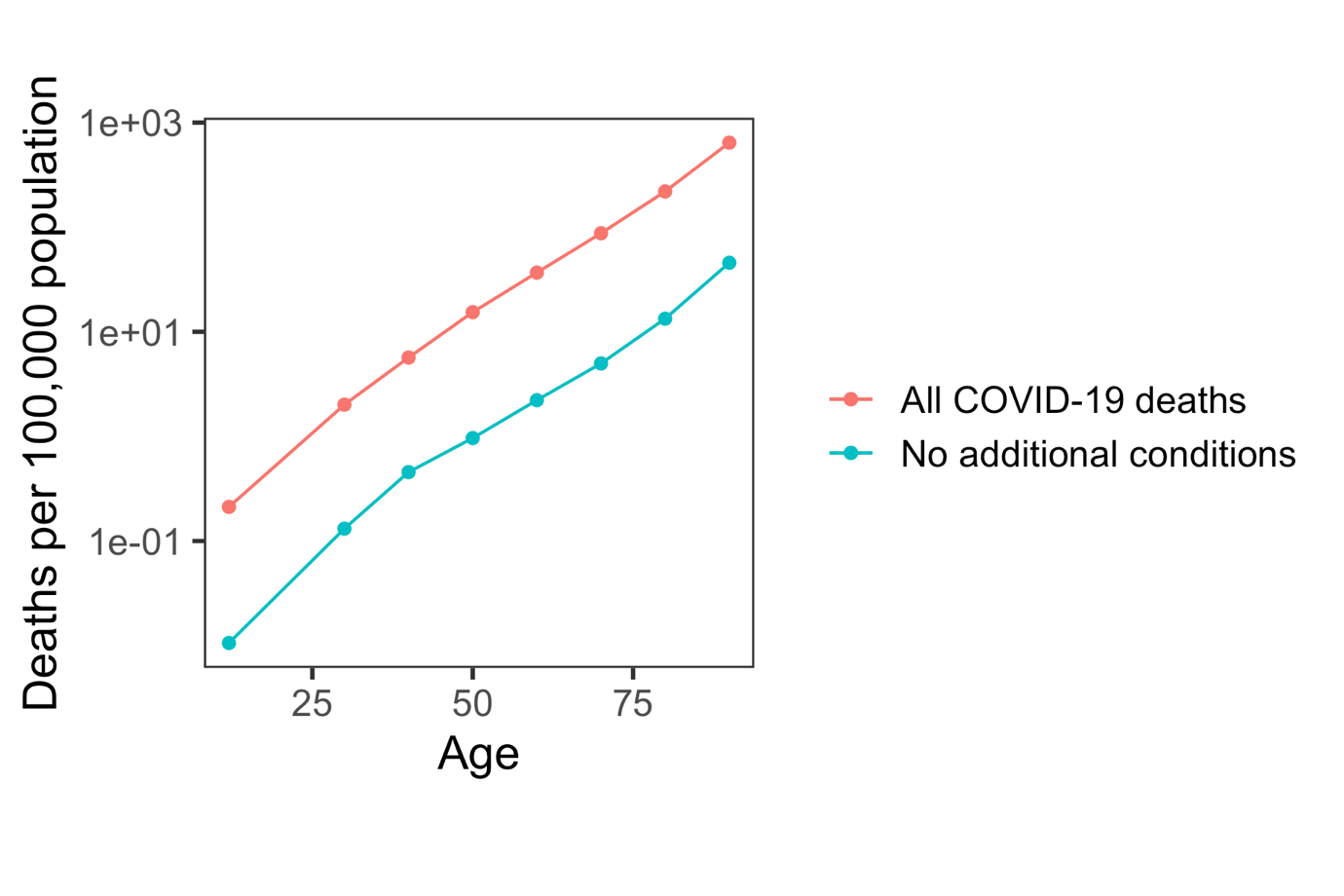
**

**Supplementary figure 6**. Deaths per 100,000 population by age in all COVID-19 deceased patients and in those who had no additional health conditions in their death certificate. COVID-19 deaths in the U.S. reported by the CDC as of 24 July, 2020. The number of deaths without conditions was obtained from the CDC. Total COVID-19 deaths: 127,940; Deaths with no additional conditions: 8,162 (6.4%).

**
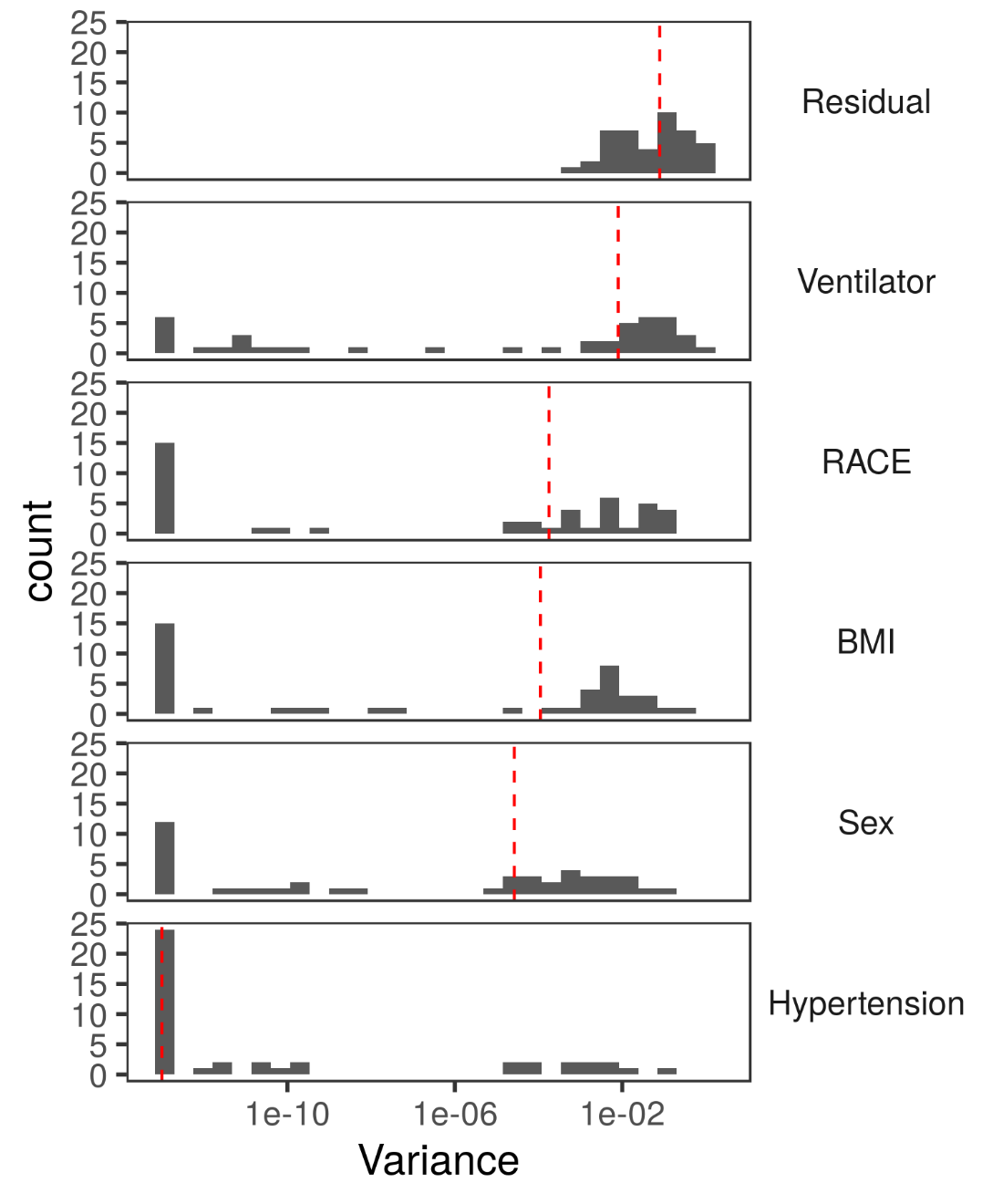
**

**Supplementary figure 7.** Distribution of variance of random effects (indicated on the right) used in the liner mixed model implemented in each tissue using log ACE2 expression and age. The median value is shown by a dashed red line. Regression coefficients of the linear mixed model in each tissue are shown in Supplementary Table 2.

**Supplementary figure 8.** Distribution of expression of ACE2 across tissues in GTEx samples. The difference between subjects with and without a ventilator at the time of death is shown. Tissues where the difference is statistically significant (Mann-Whitney, p < 0.05) are marked with an asterisk. Tissues are sorted by the combined median expression.
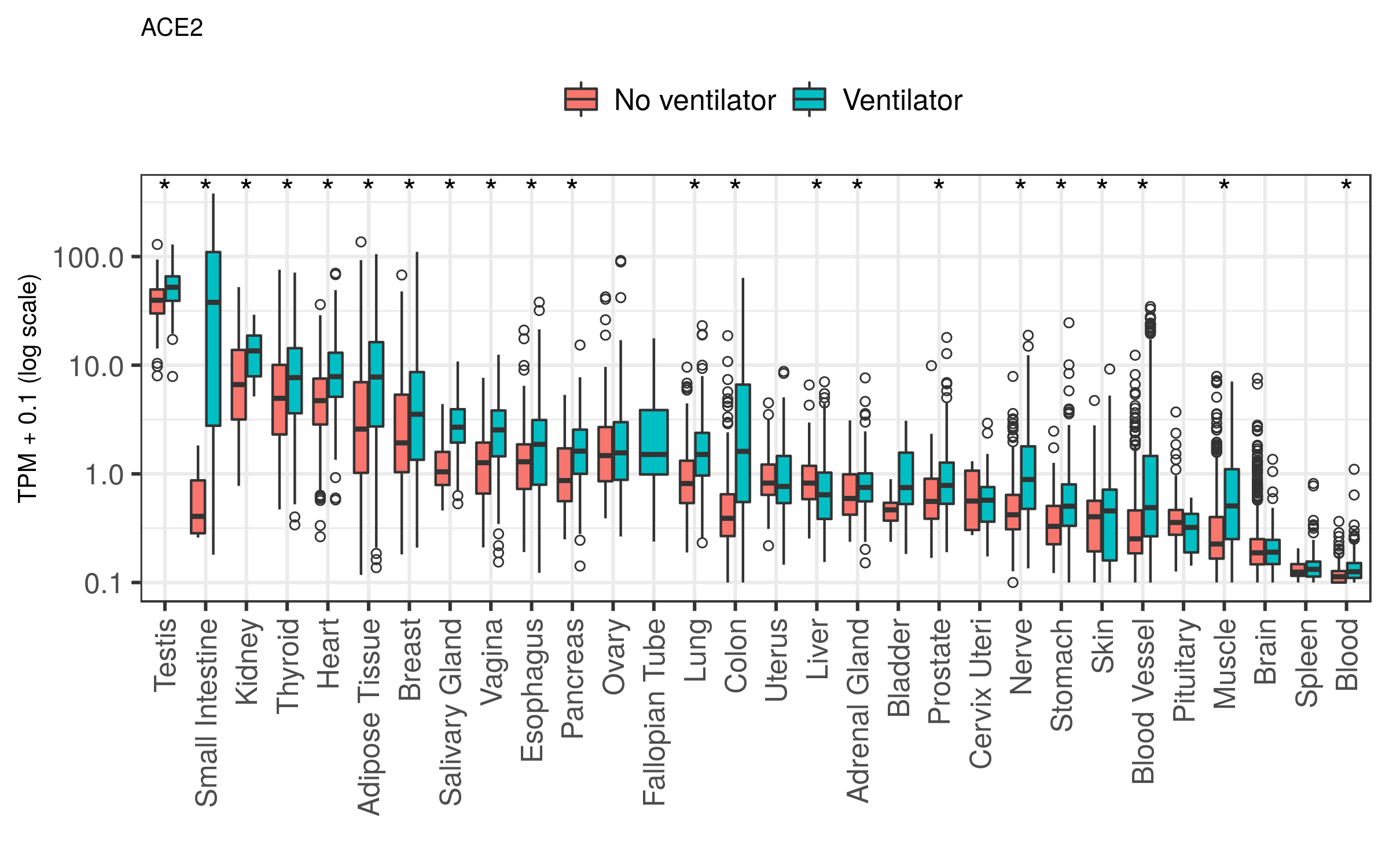


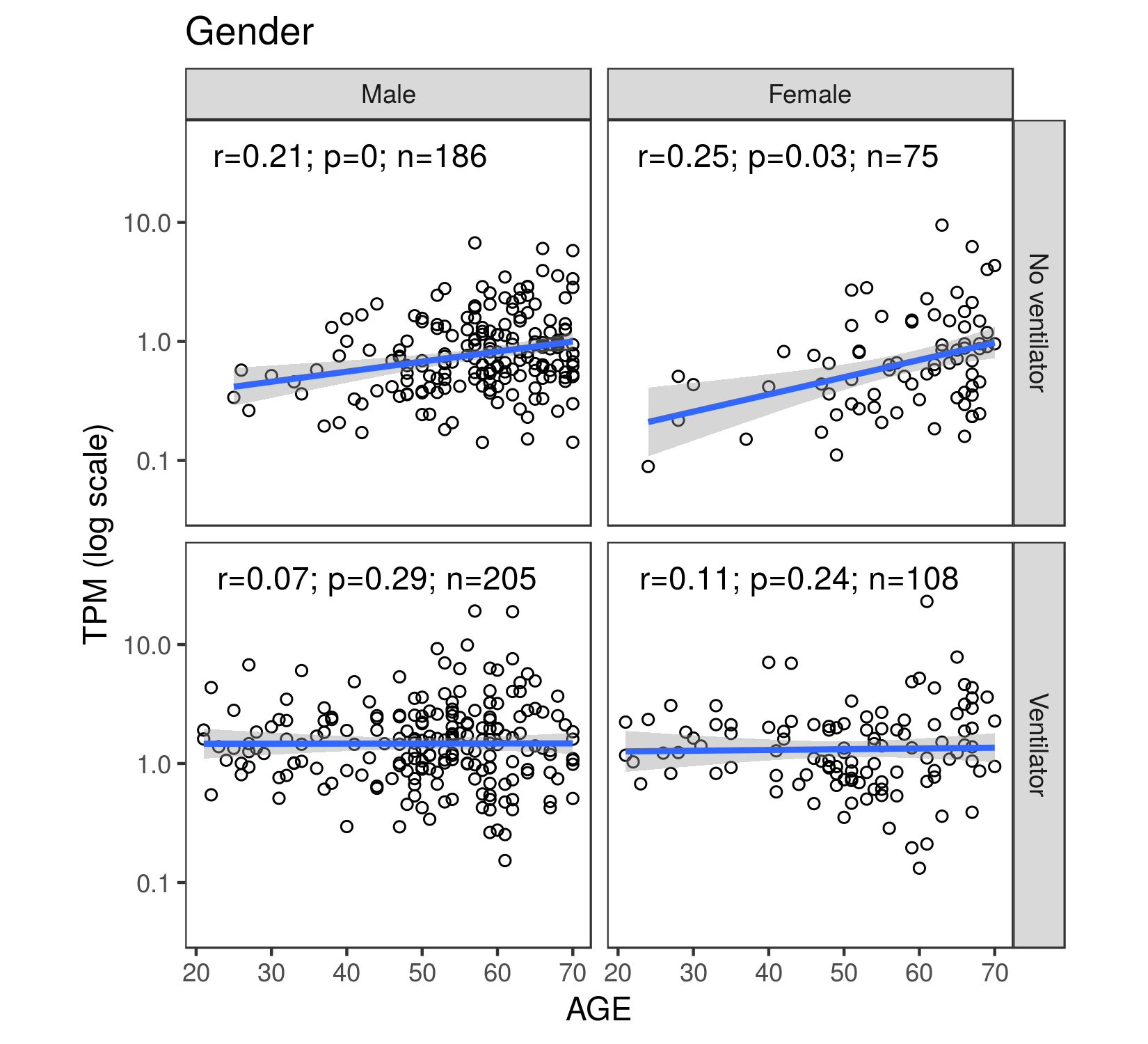


**Supplementary figure 9**. Relationship between expression of ACE2 and age in lung samples from GTEx, grouped by gender (left panels for males, and right panels for females) and by the use of a ventilator at the time of death (no ventilator in top panels, and ventilator in bottom panels). Pearson correlation coefficient (r), the corresponding p-value (p), and the number of samples (n) are indicated in each plot.


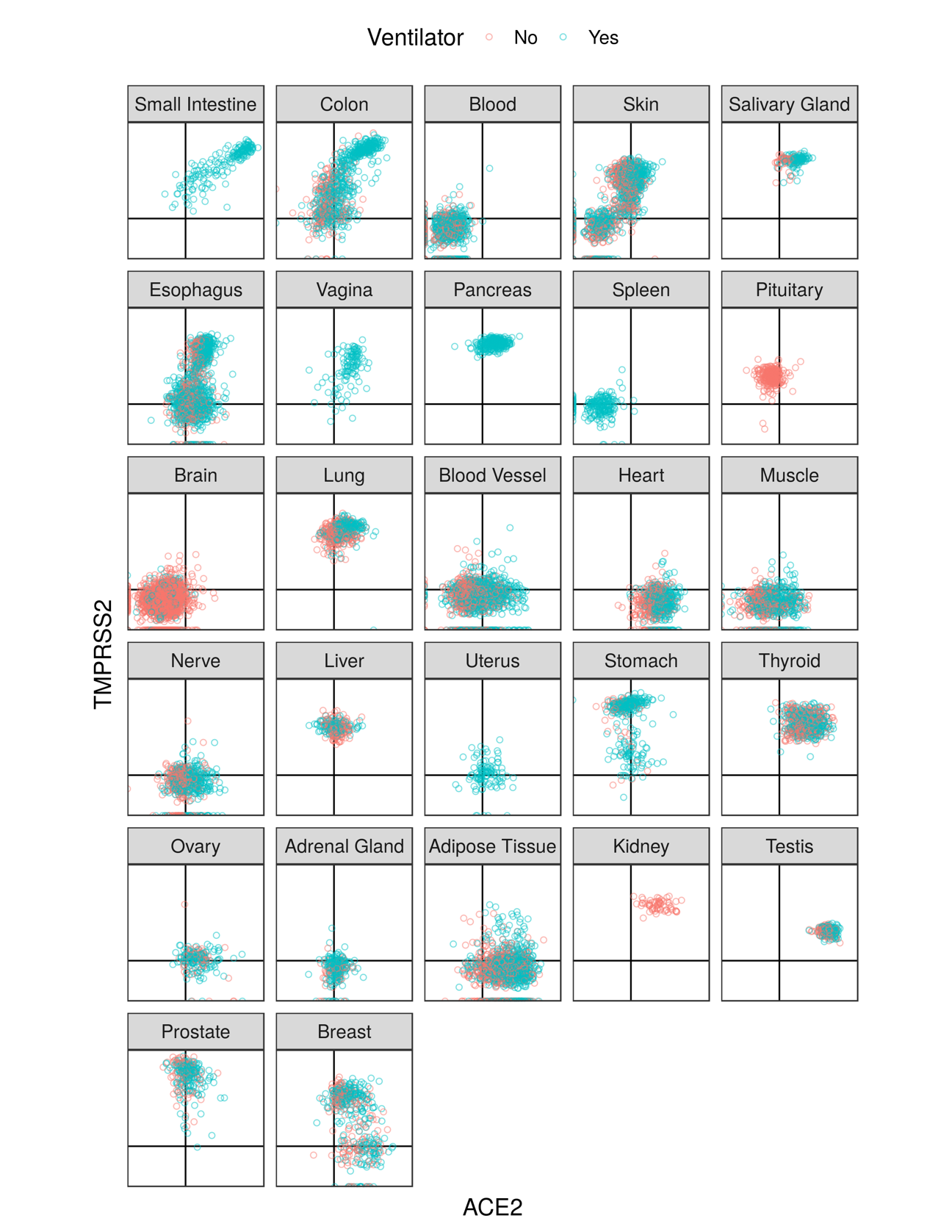


**Supplementary figure 10.** Co-expression of ACE2 (x-axis) and TMPRSS2 (y-axis) across tissues in GTEx samples. Vertical line corresponds to the median expression of ACE2 across all tissues, and the horizontal line corresponds to the median TMPRSS2 expression across all tissues. Tissues are sorted by correlation coefficient. Both axes are shown in log scale.


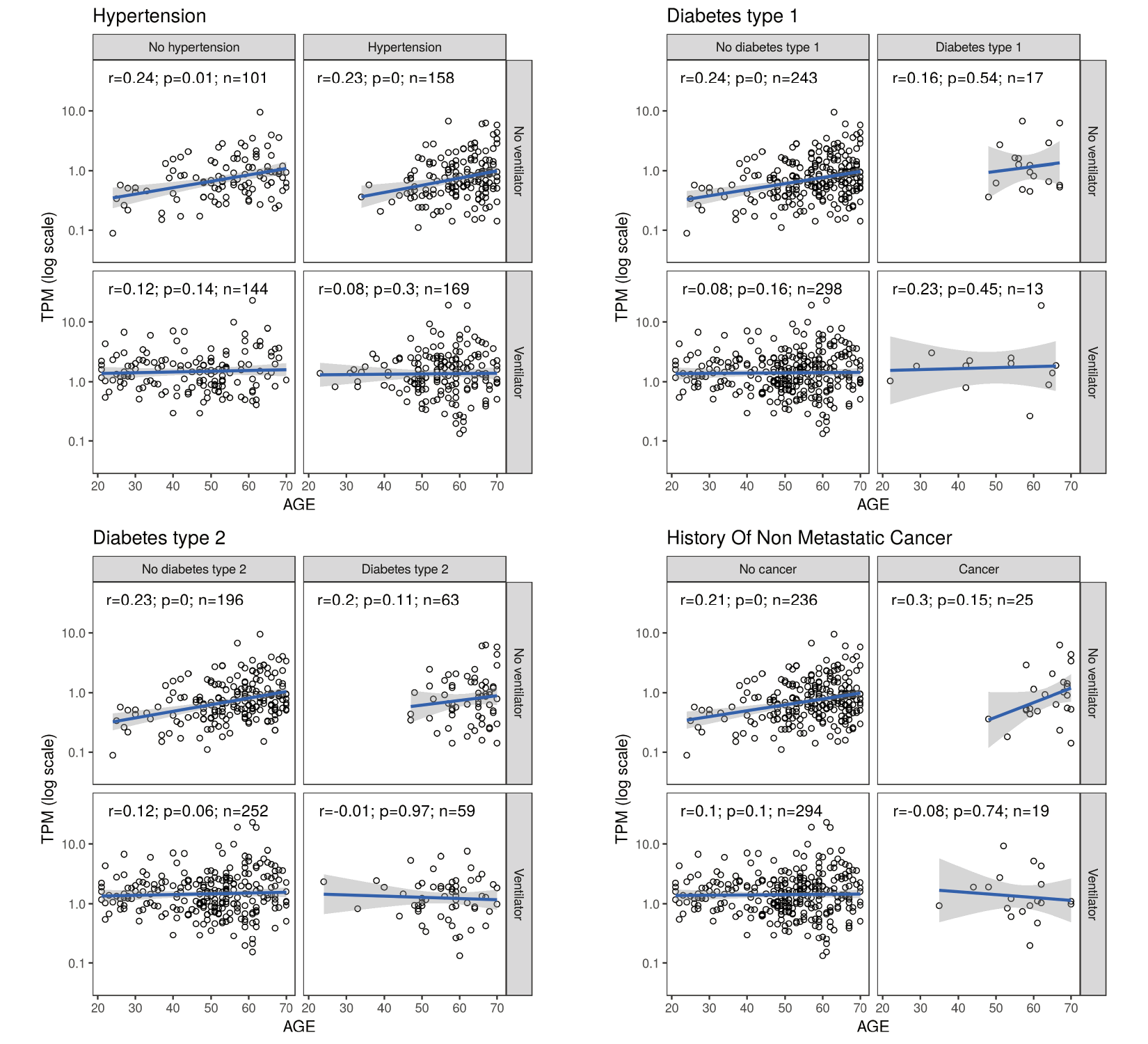


**Supplementary figure 11.** Relationship between expression of ACE2 and age in lung samples from GTEx. Samples are grouped by different subject phenotypes, including common comorbidities diagnosed in COVID-19 deceased patients: hypertension, diabetes, cancer, ischemic heart disease, renal failure, chronic respiratory disease, and cerebral cardiovascular disease. In addition, the samples are further grouped by the use of a ventilator or not at the time of death. Pearson correlation coefficient (r), the corresponding p-values (p), and the number of samples (n) are shown in each plot. Figure continues to the next page.

**Supplementary figure 11 (cont.)**


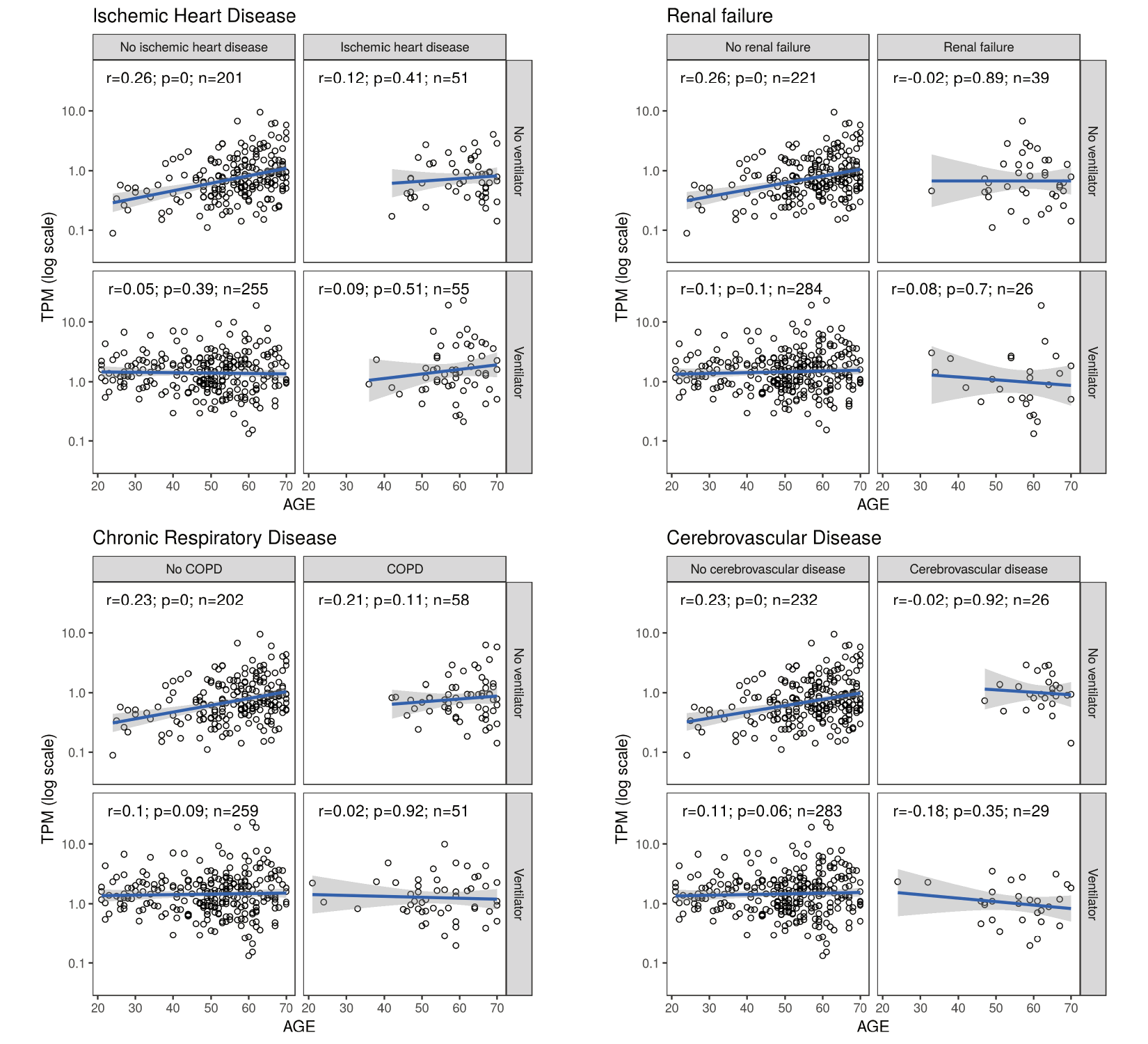


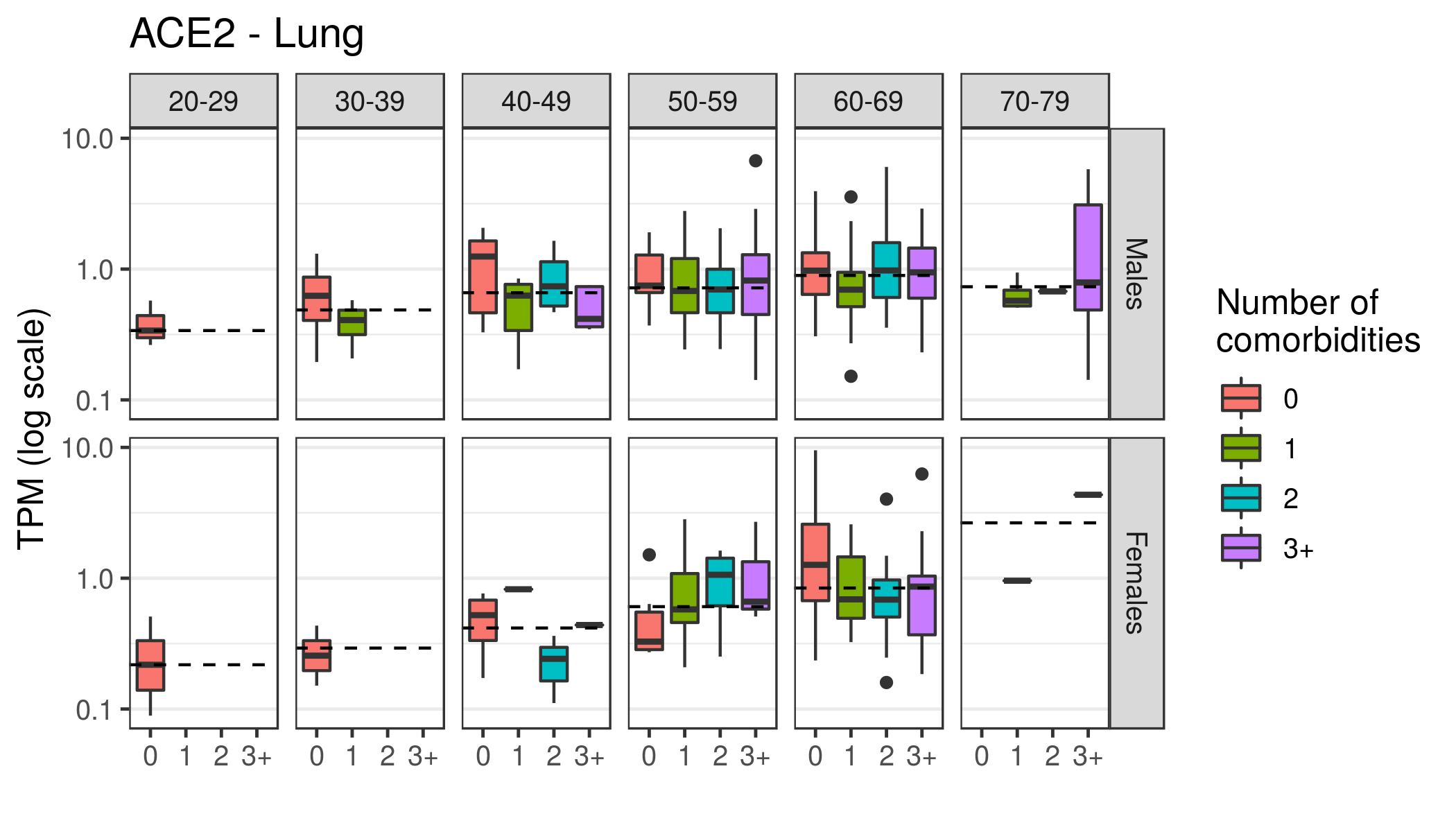


**Supplementary figure 12.** Distribution of expression of ACE2 in lung samples from subjects who were not on a ventilator at time of death. Samples were grouped in age bins, by gender, and by the number of diagnosed comorbidities. The comorbidities considered are the same as those shown in Supplementary figure 2: hypertension, ischemic heart disease, chronic respiratory disease, renal failure, cerebrovascular disease, type 1 and type 2 diabetes, and history of non-metastatic cancer. The dashed line indicates the median across samples in the same age bin and sex.


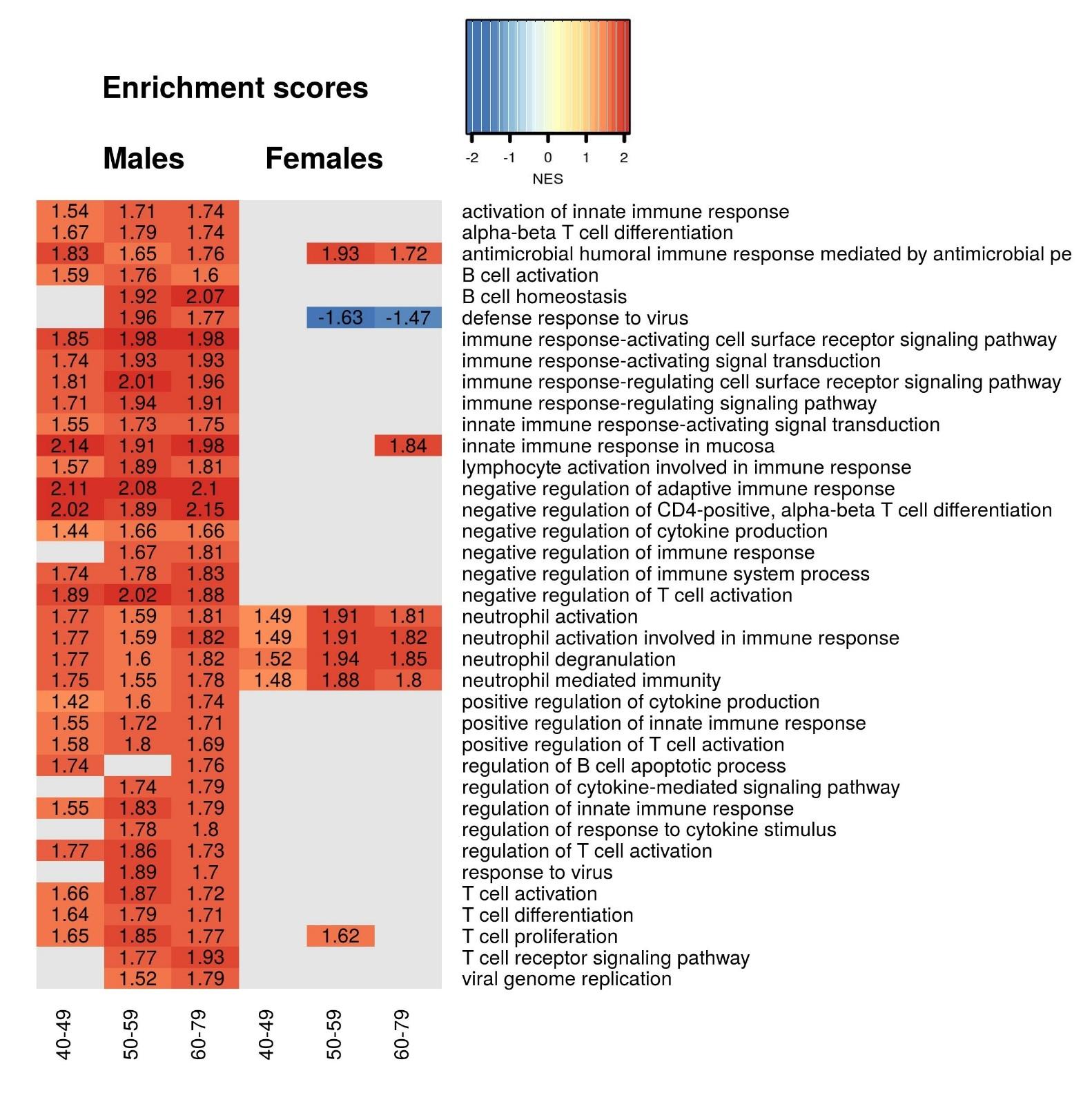


**Supplementary figure 13.** Gene ontology (GO) enrichment analysis for lung in men and women across age groups compared to young individuals for non-ventilator cases only. Samples were grouped in age bins, and each group was compared to young adults (20-39 years old). Values represent the Normalized Enrichment Score (NES) obtained from gene set enrichment analysis with following reduction for substantially overlapping pathways. Grey cells represent results below threshold for statistical significance (qvalue < 0.05).

**Supplementary figure 14.** Excess mortality from the Spanish Influenza pandemic in 1918, shown as the number of deaths per 100,000 population in the United States. Source (6).
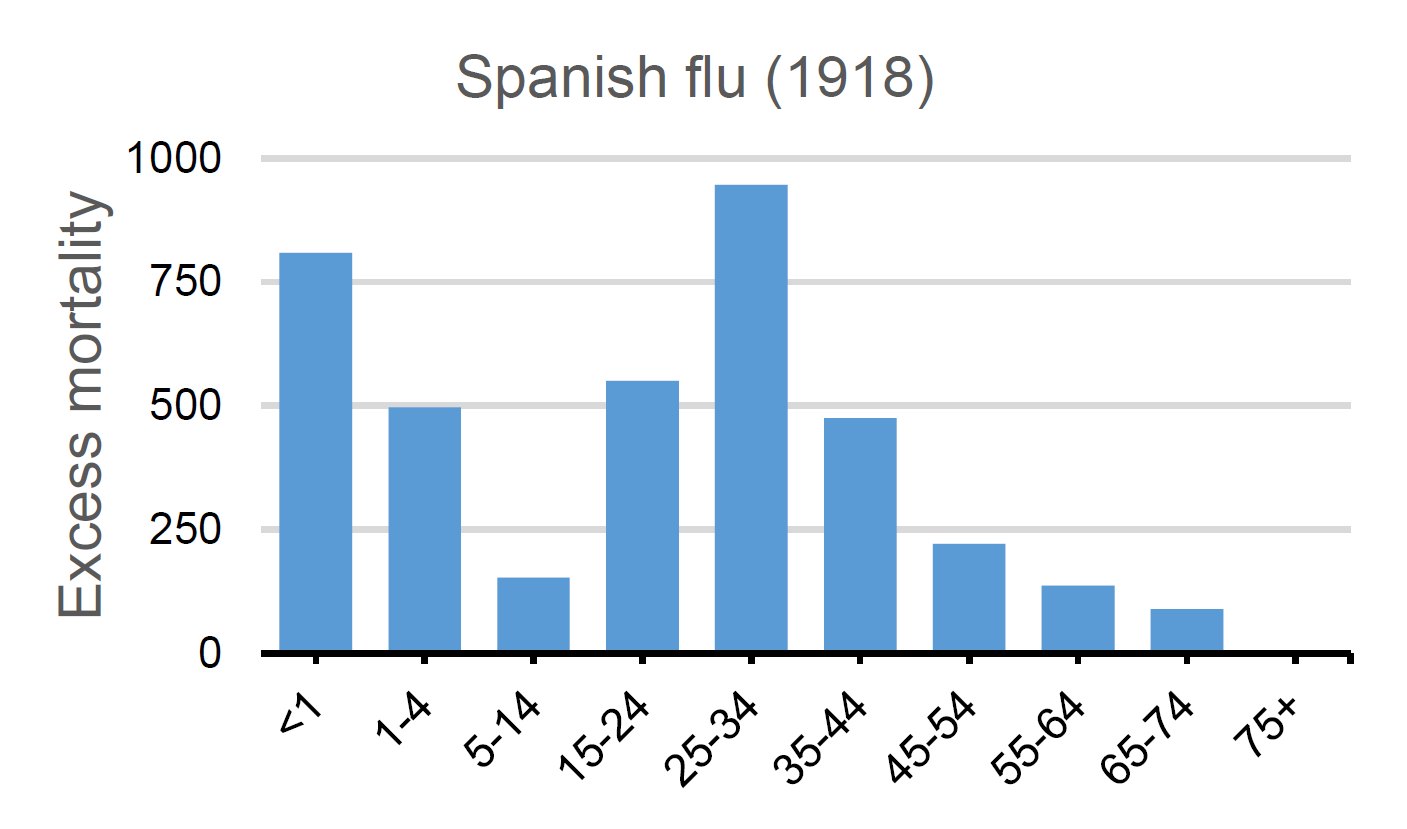


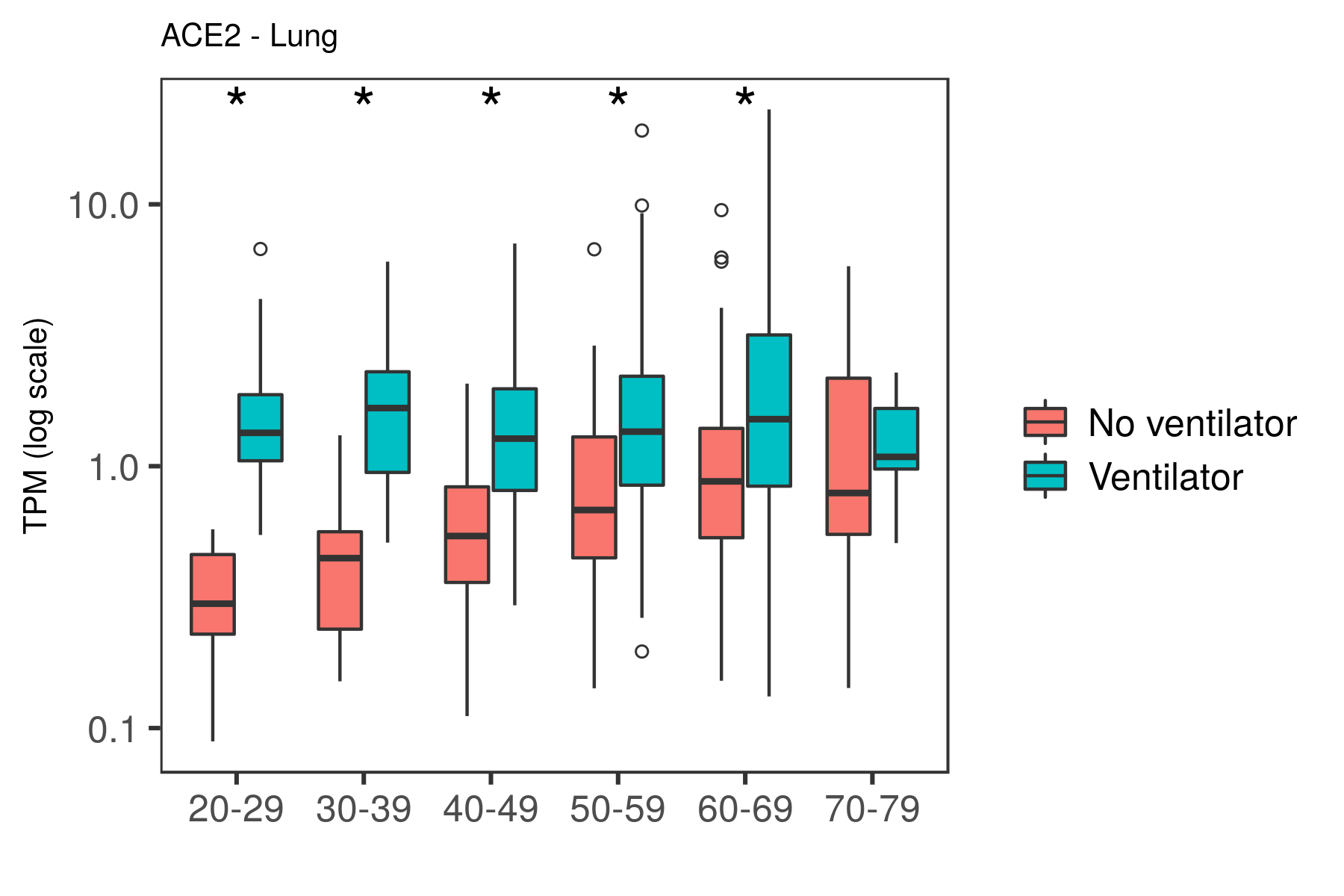


**Supplementary figure 15.** Difference in expression of ACE2 in the lung in subjects with and without ventilator, across age groups. Age groups where the difference is statistically significant (Mann-Whitney, p < 0.05) are marked with an asterisk.

**Supplementary references**

1. Istituto Superiore di Sanità - Coronavirus. https://www.epicentro.iss.it/coronavirus/. June 23, 2020.
2. Instituto de Salud Carlos III - Informes COVID-19. https://www.isciii.es/QueHacemos/Servicios/VigilanciaSaludPublicaRENAVE/EnfermedadesTransmisibles/Paginas/InformesCOVID-19.aspx. May 10, 2020.
3. Korea Center for Disease Control and Prevention (KCDC) and the Ministry of the Interior and Safety, Republic of Korea (<http://27.101.213.4/>) as of April 7, 2020.
4. Verity R, Okell LC, Dorigatti I, Winskill P, Whittaker C, Imai N, Cuomo-dannenburg G, Thompson H, Walker PGT, Fu H, Dighe A, Griffin JT, Baguelin M, Bhatia S, Boonyasiri A, Cori A, Cucunubá Z, Fitzjohn R, Gaythorpe K, Green W, Hamlet A, Hinsley W, Laydon D, Nedjati-gilani G, Riley S, Elsland S Van, Volz E, Wang H, Wang Y, Xi X, Donnelly CA, Ghani AC, and Ferguson NM. Estimates of the severity of coronavirus disease 2019: a model-based analysis. *Lancet Infect Dis* 3099: 1–9, 2020.
5. Wu Z and McGoogan JM. Characteristics of and Important Lessons from the Coronavirus Disease 2019 (COVID-19) Outbreak in China: Summary of a Report of 72314 Cases from the Chinese Center for Disease Control and Prevention. *JAMA - J Am Med Assoc*, 2020.
6. Jeffrey Luk, Peter Gross, William W. Thompson, Observations on Mortality during the 1918 Influenza Pandemic. *Clinical Infectious Diseases* 8, 1375–1378, 2001.
7. Yu G, Wang LG, Han Y, He QY. clusterProfiler: an R package for comparing biological themes among gene clusters. *OMICS* 16(5):284–287, 2012.
